# 4-(But-3-enylamino)-3-nitro­benzoic acid

**DOI:** 10.1107/S1600536809021692

**Published:** 2009-06-13

**Authors:** Shivanagere Nagojappa Narendra Babu, Aisyah Saad Abdul Rahim, Hasnah Osman, Samuel Robinson Jebas, Hoong-Kun Fun

**Affiliations:** a School of Pharmaceutical Sciences, Universiti Sains Malaysia, 11800 USM, Penang, Malaysia; b School of Chemical Sciences, Universiti Sains Malaysia, 11800 USM, Penang, Malaysia; c X-ray Crystallography Unit, School of Physics, Universiti Sains Malaysia, 11800 USM, Penang, Malaysia

## Abstract

The asymmetric unit of the title compound, C_11_H_12_N_2_O_4_, contains 12 crystallographically independent mol­ecules, labelled *A* to *L*. The nitro and carboxyl groups are twisted slightly out of the plane of the attached benzene ring in all independent mol­ecules except mol­ecules *G* and *D*. The nitro group is coplanar with the benzene ring in mol­ecule *G* and the carboxyl group is coplanar with the benzene ring in mol­ecule *D*. The orientation of the butyl group with respect to the rest of the mol­ecule is different in some of the independent mol­ecules, with the C—C—C—C torsion angles varying from 104.2 (5) to 126.6 (7)°. In each independent mol­ecule, an intra­molecular N—H⋯O hydrogen bond generates an *S*(6) ring motif. In the crystal structure, the 12 independent mol­ecules exist as six pairs of O—H⋯O hydrogen-bonded *R*_2_^2^(8) dimers. In addition, C—H⋯O hydrogen bonds are observed.

## Related literature

For heterocyclic compounds of pharmacological inter­est, see: Ishida *et al.* (2006 ▶); Kuzniewski *et al.* (2008 ▶); Wu *et al.* (2000 ▶). For bond-length data, see: Allen *et al.* (1987 ▶). For hydrogen-bond motifs, see: Bernstein *et al.* (1995 ▶). For the stability of the temperature controller used in the data collection, see: Cosier & Glazer (1986 ▶).
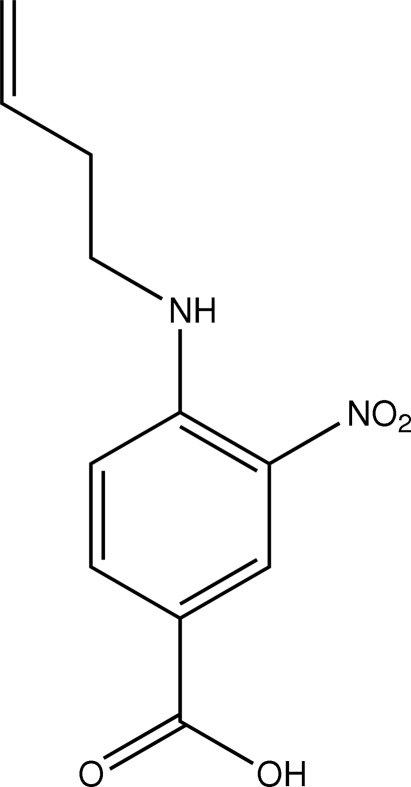


## Experimental

### 

#### Crystal data

C_11_H_12_N_2_O_4_
*M* *_r_* = 236.23
Monoclinic, 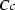

*a* = 13.3461 (5) Å
*b* = 66.777 (3) Å
*c* = 15.0195 (6) Å
β = 99.632 (2)°
*V* = 13196.9 (9) Å^3^
*Z* = 48
Mo *K*α radiation
μ = 0.11 mm^−1^
*T* = 100 K
0.33 × 0.29 × 0.13 mm

#### Data collection

Bruker SMART APEXII CCD area-detector diffractometer
Absorption correction: multi-scan (*SADABS*; Bruker, 2005 ▶) *T*_min_ = 0.965, *T*_max_ = 0.985
173518 measured reflections
21275 independent reflections
16663 reflections with *I* > 2σ(*I*)
*R*_int_ = 0.096

#### Refinement

*R*[*F* ^2^ > 2σ(*F* ^2^)] = 0.078
*wR*(*F* ^2^) = 0.213
*S* = 1.08
21275 reflections
1837 parameters
2 restraints
H-atom parameters constrained
Δρ_max_ = 0.73 e Å^−3^
Δρ_min_ = −0.38 e Å^−3^

### 

Data collection: *APEX2* (Bruker, 2005 ▶); cell refinement: *SAINT* (Bruker, 2005 ▶); data reduction: *SAINT*; program(s) used to solve structure: *SHELXTL* (Sheldrick, 2008 ▶); program(s) used to refine structure: *SHELXTL*; molecular graphics: *SHELXTL*; software used to prepare material for publication: *SHELXTL* and *PLATON* (Spek, 2009 ▶).

## Supplementary Material

Crystal structure: contains datablocks global, I. DOI: 10.1107/S1600536809021692/ci2814sup1.cif

Structure factors: contains datablocks I. DOI: 10.1107/S1600536809021692/ci2814Isup2.hkl

Additional supplementary materials:  crystallographic information; 3D view; checkCIF report

## Figures and Tables

**Table 1:** Hydrogen-bond geometry (Å, °)

*D*—H⋯*A*	*D*—H	H⋯*A*	*D*⋯*A*	*D*—H⋯*A*
N1*A*—H1*AA*⋯O1*A*	0.86	2.00	2.643 (4)	130
N1*B*—H1*BA*⋯O1*B*	0.86	1.99	2.628 (5)	130
N1*C*—H1*CA*⋯O1*C*	0.86	1.98	2.631 (4)	132
N1*D*—H1*DA*⋯O1*D*	0.86	1.99	2.634 (5)	130
N1*E*—H1*EA*⋯O1*E*	0.86	2.01	2.637 (5)	129
N1*F*—H1*FA*⋯O1*F*	0.86	2.00	2.641 (4)	130
N1*G*—H1*GA*⋯O1*G*	0.86	1.98	2.624 (5)	130
N1*H*—H1*HA*⋯O1*H*	0.86	1.97	2.621 (5)	131
N1*I*—H1*IA*⋯O1*I*	0.86	1.99	2.632 (4)	130
N1*J*—H1*JA*⋯O1*J*	0.86	1.99	2.627 (4)	131
N1*K*—H1*KA*⋯O1*K*	0.86	1.96	2.615 (4)	132
N1*L*—H1*LA*⋯O1*L*	0.86	2.01	2.645 (4)	130
O3*A*—H3*AB*⋯O4*C*	0.82	1.80	2.612 (5)	176
O3*C*—H3*CB*⋯O4*A*	0.82	1.82	2.630 (4)	172
O3*B*—H3*BB*⋯O4*L*	0.82	1.81	2.626 (5)	173
O3*L*—H3*LB*⋯O4*B*	0.82	1.80	2.613 (5)	169
O3*D*—H3*DB*⋯O4*J*	0.82	1.80	2.618 (5)	173
O3*J*—H3*JB*⋯O4*D*	0.82	1.81	2.626 (5)	175
O3*E*—H3*EB*⋯O4*I*	0.82	1.81	2.624 (5)	170
O3*I*—H3*IB*⋯O4*E*	0.82	1.82	2.635 (5)	174
O3*G*—H3*GB*⋯O4*K*	0.82	1.80	2.616 (5)	172
O3*K*—H3*KB*⋯O4*G*	0.82	1.83	2.633 (5)	163
O3*F*—H3*FB*⋯O4*H*^i^	0.82	1.79	2.605 (5)	172
O3*H*—H3*HB*⋯O4*F*^ii^	0.82	1.82	2.611 (5)	164
C6*B*—H6*BA*⋯O2*H*^i^	0.93	2.45	3.169 (5)	134
C6*H*—H6*HA*⋯O2*B*^iii^	0.93	2.55	3.243 (5)	131
C6*I*—H6*IA*⋯O2*C*^ii^	0.93	2.53	3.218 (5)	131
C6*K*—H6*KA*⋯O2*K*^iv^	0.93	2.40	3.149 (4)	138
C9*C*—H9*CB*⋯O1*B*	0.97	2.59	3.477 (6)	152

Symmetry codes: (i) 
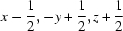
; (ii) 
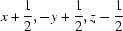
; (iii) 
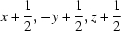
; (iv) 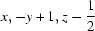.

**Fig. 1.**
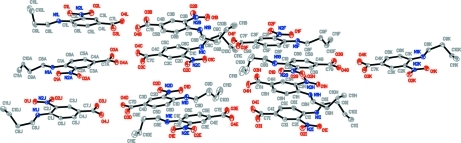
The twelve independent molecules in the asymmetric unit of the title compound, showing the atom-numbering scheme. Displacement ellipsoids are drawn at the 50% probability level. H atoms have been omitted for clarity.

**Fig. 2.**
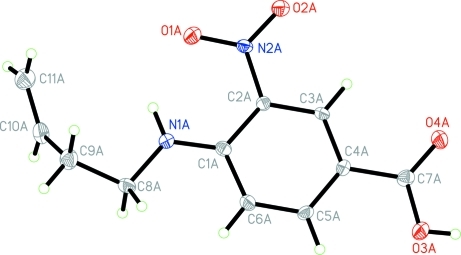
One of the twelve independent molecules in the asymmetric unit to show clearly the atomic labelling. The other eleven molecules are labelled in a similar manner.

**Fig. 3.**
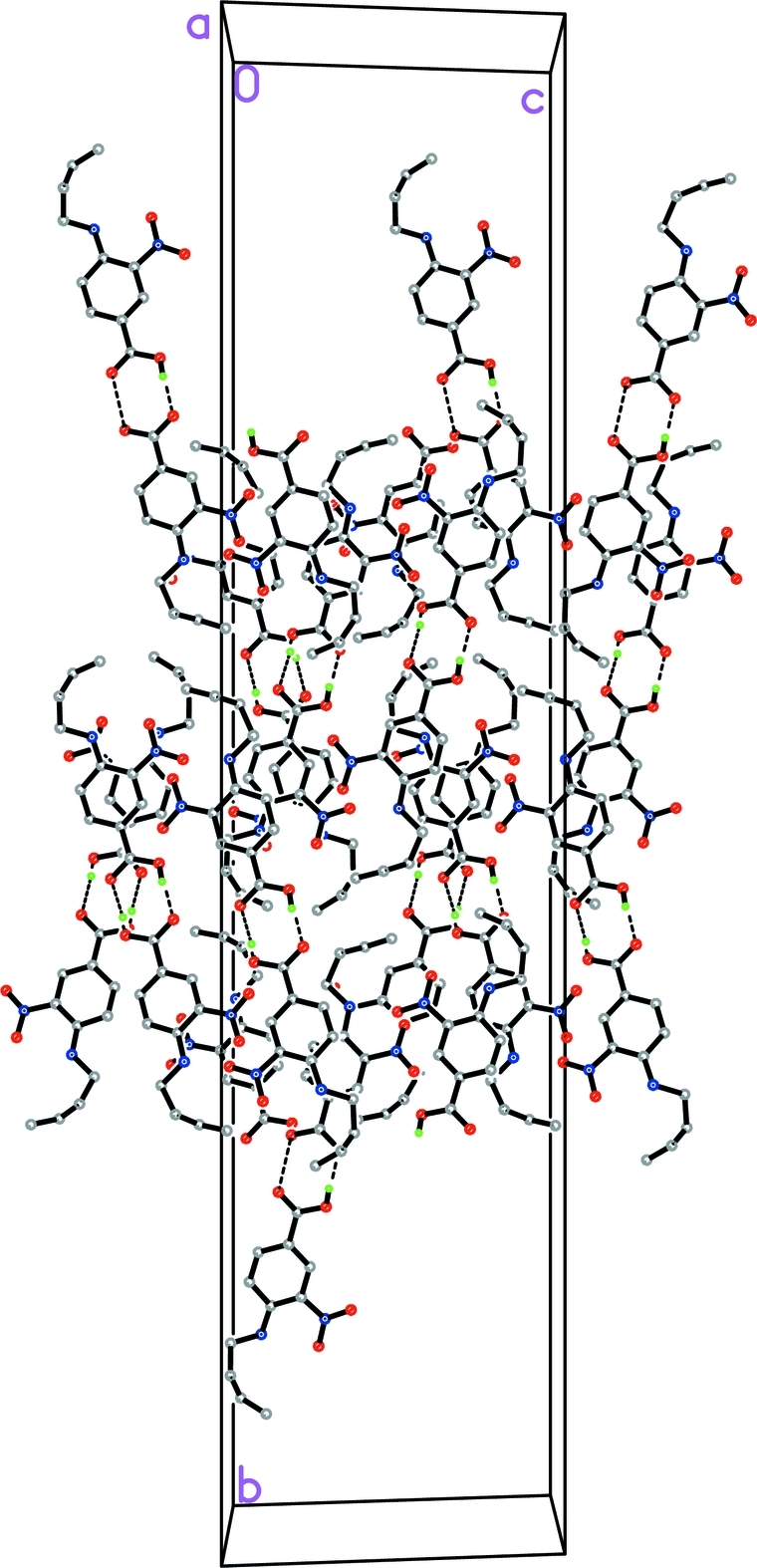
The crystal packing of the title compound, showing hydrogen-bonded (dashed lines) dimers.

## supplementary crystallographic information

### Comment

Various heterocyclic compounds of pharmacological interests (Ishida *et al.*, 2006; Kuzniewski *et al.*, 2008; Wu *et al.*, 2000) can be synthesized by nitro benzoic acid derivatives as the precursors. We report here the crystal structure of the title compound, which was synthesized as an intermediate.

The asymmetric unit of the title compound (Fig. 1) comprises of twelve crystallographically independent molecules labelled as A to L. The bond lengths (Allen *et al.*, 1987) and bond angles are normal. The nitro group (N2/O1/O2) is twisted away from the attached benzene ring (C1-C6) in eleven molecules forming dihedral angles of 1.54 (1)° in molecule A [5.45 (2)° in B; 4.68 (2)° in C; 2.44 (1) in D; 5.10 (2)° in E; 5.84 (2)° in F; 3.29 (2)° in H; 6.96 (2)° in I; 7.48 (2)° in J; 2.44 (2)° in K and 4.67 (2)° in L]. In molecule G, the nitro group (N2/O1/O2) is almost coplanar with the attached benzene ring (C1-C6) forming a dihedral angle of 0.99 (2)°. The dihedral angles formed by the carboxyl groups (C7/O3/O4) with the attached benzene rings are 5.24 (1)° in molecule A [4.49 (2)° in B; 1.69 (2)° in C; 19.79 (2)° in E; 18.38 (2)° in F; 16.46 (3)° in G; 15.11 (2)° in H; 27.26 (2)° in I; 28.61 (2)° in J; 16.39 (2)° in K; 21.94 (2)° in *L*], indicating that they are twisted from the mean plane of the benzene ring. In molecule D, the carboxyl group is coplanar with the benzene ring [dihedral angle 0.26 (1)°]. The orientation of the butyl group of the but-3-enylamino unit (N1/C8-C11) is different in some of the independent molecules. The C8-C9-C10-C11 torsion angle is -121.7 (5)° in molecule A [-109.4 (6)° in B; -107.0 (6)° in C; 126.3 (7)° in D; 123.7 (5)° in E; 123.0 (5)° in F; 126.6 (7)° in G; -104.2 (5)° in H; -112.9 (5)° in I; 111.6 (5)° in J; -104.8 (5)° in K and 121.8 (6)° in L]. In each independent molecule, an intramolecular N—H···O hydrogen bond generates an S(6) ring motif (Bernstein *et al.*, 1995).

In the crystal structure, the twelve independent molecules exist as six pairs of O—H···O hydrogen-bonded *R*_2_^2^(8) dimers (Table 1 and Fig. 3). In addition, C—H···O hydrogen bonds are observed.

### Experimental

A mixture of ethyl 4-(but-3-enylamino)-3-nitro-benzoate (0.25 g, 0.001 mol) and KOH (0.112 g, 0.002 mol) was refluxed in aqueous ethanol (5 ml) for 3 h. After completion of the reaction, ethanol was distilled off and the reaction mixture was diluted with water (5 ml). The aqueous layer was washed with dichloromethane (5 ml × 2) and acidified with concentrated HCl to afford a yellow solid. Yellow single crystals were obtained after recrystallization of the crude product with hot ethyl acetate.

### Refinement

H atoms were positioned geometrically [C-H = 0.93–0.97 Å; N-H = 0.86Å and O-H = 0.82 Å] and refined using a riding model with *U*_iso_(H) = 1.2*U*_eq_(C,N) and 1.5*U*_eq_(O). In the absence of significant anomalous scattering effects, 20703 Friedel pairs were averaged.

### Crystal data

**Table:**

C_11_H_12_N_2_O_4_	*F*(000) = 5952
*M**_r_* = 236.23	*D*_x_ = 1.427 Mg m^−^^3^
Monoclinic, *C**c*	Mo *K*α radiation, λ = 0.71073 Å
Hall symbol: C -2yc	Cell parameters from 9973 reflections
*a* = 13.3461 (5) Å	θ = 2.7–33.0°
*b* = 66.777 (3) Å	µ = 0.11 mm^−^^1^
*c* = 15.0195 (6) Å	*T* = 100 K
β = 99.632 (2)°	Block, yellow
*V* = 13196.9 (9) Å^3^	0.33 × 0.29 × 0.13 mm
*Z* = 48	

### Data collection

**Table:**

Bruker SMART APEXII CCD area-detector diffractometer	21275 independent reflections
Radiation source: fine-focus sealed tube	16663 reflections with *I* > 2σ(*I*)
graphite	*R*_int_ = 0.096
φ and ω scans	θ_max_ = 31.1°, θ_min_ = 0.6°
Absorption correction: multi-scan (*SADABS*; Bruker, 2005)	*h* = −19→19
*T*_min_ = 0.965, *T*_max_ = 0.985	*k* = −96→96
173518 measured reflections	*l* = −21→21

### Refinement

**Table:**

Refinement on *F*^2^	Primary atom site location: structure-invariant direct methods
Least-squares matrix: full	Secondary atom site location: difference Fourier map
*R*[*F*^2^ > 2σ(*F*^2^)] = 0.078	Hydrogen site location: inferred from neighbouring sites
*wR*(*F*^2^) = 0.213	H-atom parameters constrained
*S* = 1.08	*w* = 1/[σ^2^(*F*_o_^2^) + (0.1104*P*)^2^ + 13.1603*P*] where *P* = (*F*_o_^2^ + 2*F*_c_^2^)/3
21275 reflections	(Δ/σ)_max_ = 0.003
1837 parameters	Δρ_max_ = 0.73 e Å^−^^3^
2 restraints	Δρ_min_ = −0.37 e Å^−^^3^

### Special details

**Table:**

Experimental. The crystal was placed in the cold stream of an Oxford Cyrosystems Cobra open-flow nitrogen cryostat (Cosier & Glazer, 1986) operating at 100.0 (1) K.
Geometry. All e.s.d.'s (except the e.s.d. in the dihedral angle between two l.s. planes) are estimated using the full covariance matrix. The cell e.s.d.'s are taken into account individually in the estimation of e.s.d.'s in distances, angles and torsion angles; correlations between e.s.d.'s in cell parameters are only used when they are defined by crystal symmetry. An approximate (isotropic) treatment of cell e.s.d.'s is used for estimating e.s.d.'s involving l.s. planes.
Refinement. Refinement of *F*^2^ against ALL reflections. The weighted *R*-factor *wR* and goodness of fit *S* are based on *F*^2^, conventional *R*-factors *R* are based on *F*, with *F* set to zero for negative *F*^2^. The threshold expression of *F*^2^ > σ(*F*^2^) is used only for calculating *R*-factors(gt) *etc*. and is not relevant to the choice of reflections for refinement. *R*-factors based on *F*^2^ are statistically about twice as large as those based on *F*, and *R*- factors based on ALL data will be even larger.

### Fractional atomic coordinates and isotropic or equivalent isotropic displacement parameters (Å^2^)

**Table:**

	*x*	*y*	*z*	*U*_iso_*/*U*_eq_	
O1A	0.4798 (2)	−0.00716 (5)	0.5335 (2)	0.0206 (6)	
O2A	0.4840 (2)	0.02449 (5)	0.5645 (2)	0.0198 (6)	
O3A	0.4556 (2)	0.06616 (5)	0.1916 (2)	0.0225 (6)	
H3AB	0.4501	0.0782	0.1819	0.034*	
O4A	0.4515 (2)	0.07584 (4)	0.3347 (2)	0.0200 (6)	
N1A	0.4905 (3)	−0.01972 (5)	0.3685 (2)	0.0154 (6)	
H1AA	0.4904	−0.0234	0.4234	0.018*	
N2A	0.4804 (2)	0.01073 (5)	0.5092 (2)	0.0139 (6)	
C1A	0.4820 (3)	−0.00012 (6)	0.3488 (3)	0.0135 (7)	
C2A	0.4790 (3)	0.01539 (6)	0.4155 (3)	0.0127 (7)	
C3A	0.4705 (3)	0.03555 (6)	0.3914 (3)	0.0128 (7)	
H3AA	0.4675	0.0452	0.4355	0.015*	
C4A	0.4666 (3)	0.04124 (6)	0.3024 (3)	0.0136 (7)	
C5A	0.4696 (3)	0.02653 (6)	0.2357 (3)	0.0142 (7)	
H5AA	0.4658	0.0303	0.1756	0.017*	
C6A	0.4781 (3)	0.00665 (6)	0.2594 (3)	0.0157 (7)	
H6AA	0.4814	−0.0028	0.2144	0.019*	
C7A	0.4571 (3)	0.06271 (6)	0.2777 (3)	0.0163 (7)	
C8A	0.4999 (3)	−0.03516 (6)	0.3019 (3)	0.0170 (7)	
H8AA	0.5488	−0.0310	0.2644	0.020*	
H8AB	0.4349	−0.0372	0.2631	0.020*	
C9A	0.5352 (3)	−0.05477 (6)	0.3505 (3)	0.0225 (8)	
H9AA	0.5494	−0.0645	0.3063	0.027*	
H9AB	0.5978	−0.0523	0.3921	0.027*	
C10A	0.4590 (4)	−0.06310 (6)	0.4006 (3)	0.0235 (9)	
H10A	0.3953	−0.0659	0.3674	0.028*	
C11A	0.4722 (4)	−0.06695 (7)	0.4881 (3)	0.0264 (9)	
H11A	0.5346	−0.0644	0.5240	0.032*	
H11B	0.4192	−0.0722	0.5136	0.032*	
O1B	0.1456 (3)	0.20916 (5)	0.1093 (2)	0.0339 (8)	
O2B	0.1513 (3)	0.18594 (5)	0.0112 (2)	0.0290 (7)	
O3B	0.1853 (3)	0.11707 (5)	0.0853 (2)	0.0309 (8)	
H3BB	0.1951	0.1051	0.0773	0.046*	
O4B	0.2002 (3)	0.10711 (5)	0.2301 (2)	0.0338 (8)	
N1B	0.1869 (3)	0.20140 (6)	0.2831 (3)	0.0266 (8)	
H1BA	0.1717	0.2107	0.2434	0.032*	
N2B	0.1537 (3)	0.19127 (6)	0.0896 (3)	0.0259 (8)	
C1B	0.1829 (3)	0.18234 (7)	0.2541 (3)	0.0227 (9)	
C2B	0.1676 (3)	0.17665 (7)	0.1610 (3)	0.0218 (8)	
C3B	0.1700 (3)	0.15687 (7)	0.1344 (3)	0.0220 (8)	
H3BA	0.1624	0.1537	0.0733	0.026*	
C4B	0.1837 (3)	0.14176 (7)	0.1985 (3)	0.0247 (9)	
C5B	0.1965 (3)	0.14686 (7)	0.2908 (3)	0.0240 (9)	
H5BA	0.2060	0.1368	0.3343	0.029*	
C6B	0.1952 (3)	0.16654 (7)	0.3173 (3)	0.0253 (9)	
H6BA	0.2027	0.1695	0.3786	0.030*	
C7B	0.1905 (3)	0.12053 (7)	0.1718 (3)	0.0246 (9)	
C8B	0.2151 (4)	0.20750 (8)	0.3773 (3)	0.0308 (10)	
H8BA	0.2706	0.1992	0.4069	0.037*	
H8BB	0.1578	0.2057	0.4086	0.037*	
C9B	0.2469 (4)	0.22914 (8)	0.3813 (4)	0.0347 (11)	
H9BA	0.3024	0.2309	0.3478	0.042*	
H9BB	0.2712	0.2329	0.4436	0.042*	
C10B	0.1595 (4)	0.24261 (7)	0.3423 (3)	0.0286 (10)	
H10B	0.1066	0.2442	0.3749	0.034*	
C11B	0.1537 (5)	0.25217 (9)	0.2657 (4)	0.0429 (13)	
H11C	0.2055	0.2509	0.2316	0.051*	
H11D	0.0978	0.2603	0.2453	0.051*	
O1C	0.4371 (3)	0.20715 (5)	0.2700 (2)	0.0309 (8)	
O2C	0.4437 (3)	0.18319 (5)	0.3684 (2)	0.0276 (7)	
O3C	0.4531 (3)	0.11428 (5)	0.2970 (2)	0.0254 (7)	
H3CB	0.4570	0.1022	0.3063	0.038*	
O4C	0.4392 (3)	0.10425 (5)	0.1523 (2)	0.0272 (7)	
N1C	0.4024 (3)	0.19865 (6)	0.0965 (2)	0.0222 (7)	
H1CA	0.4072	0.2079	0.1367	0.027*	
N2C	0.4381 (3)	0.18899 (6)	0.2901 (3)	0.0222 (7)	
C1C	0.4165 (3)	0.17956 (6)	0.1254 (3)	0.0200 (8)	
C2C	0.4323 (3)	0.17402 (6)	0.2187 (3)	0.0181 (7)	
C3C	0.4406 (3)	0.15417 (6)	0.2457 (3)	0.0181 (7)	
H3CA	0.4494	0.1511	0.3069	0.022*	
C4C	0.4362 (3)	0.13885 (6)	0.1829 (3)	0.0184 (7)	
C5C	0.4237 (3)	0.14409 (6)	0.0905 (3)	0.0190 (8)	
H5CA	0.4219	0.1340	0.0474	0.023*	
C6C	0.4144 (3)	0.16358 (7)	0.0631 (3)	0.0206 (8)	
H6CA	0.4064	0.1665	0.0018	0.025*	
C7C	0.4439 (3)	0.11772 (6)	0.2093 (3)	0.0203 (8)	
C8C	0.3795 (4)	0.20470 (7)	0.0011 (3)	0.0235 (9)	
H8CA	0.3304	0.1956	−0.0321	0.028*	
H8CB	0.4409	0.2043	−0.0255	0.028*	
C9C	0.3365 (4)	0.22596 (7)	−0.0039 (3)	0.0257 (9)	
H9CA	0.3147	0.2296	−0.0667	0.031*	
H9CB	0.2774	0.2263	0.0258	0.031*	
C10C	0.4126 (4)	0.24106 (7)	0.0396 (3)	0.0266 (9)	
H10C	0.4656	0.2444	0.0093	0.032*	
C11C	0.4099 (5)	0.24996 (8)	0.1176 (4)	0.0392 (12)	
H11E	0.3580	0.2470	0.1499	0.047*	
H11F	0.4598	0.2592	0.1404	0.047*	
O1D	0.6730 (3)	0.17158 (5)	0.3481 (2)	0.0273 (7)	
O2D	0.6851 (3)	0.13975 (5)	0.3179 (2)	0.0263 (7)	
O3D	0.7302 (3)	0.09808 (5)	0.6904 (2)	0.0242 (6)	
H3DB	0.7386	0.0860	0.6997	0.036*	
O4D	0.7298 (2)	0.08858 (5)	0.5475 (2)	0.0211 (6)	
N1D	0.6674 (3)	0.18377 (5)	0.5140 (3)	0.0239 (8)	
H1DA	0.6628	0.1875	0.4586	0.029*	
N2D	0.6820 (3)	0.15353 (5)	0.3716 (2)	0.0193 (7)	
C1D	0.6813 (3)	0.16414 (6)	0.5324 (3)	0.0191 (8)	
C2D	0.6873 (3)	0.14896 (7)	0.4670 (3)	0.0185 (8)	
C3D	0.7010 (3)	0.12866 (6)	0.4903 (3)	0.0165 (7)	
H3DA	0.7041	0.1191	0.4457	0.020*	
C4D	0.7096 (3)	0.12290 (6)	0.5797 (3)	0.0176 (7)	
C5D	0.7035 (3)	0.13769 (7)	0.6457 (3)	0.0197 (8)	
H5DA	0.7093	0.1339	0.7058	0.024*	
C6D	0.6893 (3)	0.15745 (7)	0.6238 (3)	0.0199 (8)	
H6DA	0.6847	0.1667	0.6692	0.024*	
C7D	0.7249 (3)	0.10172 (6)	0.6044 (3)	0.0191 (8)	
C8D	0.6595 (4)	0.19921 (7)	0.5817 (3)	0.0296 (10)	
H8DA	0.7264	0.2022	0.6153	0.036*	
H8DB	0.6171	0.1945	0.6238	0.036*	
C9D	0.6126 (5)	0.21821 (8)	0.5334 (4)	0.0446 (14)	
H9DA	0.5486	0.2149	0.4954	0.054*	
H9DB	0.5991	0.2280	0.5778	0.054*	
C10D	0.6831 (5)	0.22691 (9)	0.4773 (4)	0.0415 (13)	
H10D	0.7494	0.2296	0.5049	0.050*	
C11D	0.6572 (6)	0.23101 (10)	0.3901 (4)	0.0476 (15)	
H11G	0.5915	0.2284	0.3607	0.057*	
H11H	0.7048	0.2365	0.3584	0.057*	
O1E	0.9368 (3)	0.16267 (5)	0.5327 (2)	0.0292 (7)	
O2E	0.9200 (3)	0.19429 (5)	0.5618 (2)	0.0319 (8)	
O3E	0.8969 (3)	0.23643 (5)	0.1892 (2)	0.0280 (7)	
H3EB	0.8920	0.2485	0.1804	0.042*	
O4E	0.9056 (3)	0.24640 (5)	0.3334 (2)	0.0277 (7)	
N1E	0.9343 (3)	0.15003 (6)	0.3660 (3)	0.0224 (7)	
H1EA	0.9378	0.1462	0.4211	0.027*	
N2E	0.9277 (3)	0.18051 (6)	0.5081 (3)	0.0239 (8)	
C1E	0.9296 (3)	0.16985 (7)	0.3479 (3)	0.0202 (8)	
C2E	0.9259 (3)	0.18532 (7)	0.4136 (3)	0.0206 (8)	
C3E	0.9181 (3)	0.20535 (7)	0.3901 (3)	0.0224 (8)	
H3EA	0.9154	0.2149	0.4345	0.027*	
C4E	0.9143 (3)	0.21133 (7)	0.3012 (3)	0.0245 (9)	
C5E	0.9188 (3)	0.19634 (7)	0.2340 (3)	0.0228 (9)	
H5EA	0.9165	0.2001	0.1741	0.027*	
C6E	0.9265 (3)	0.17648 (7)	0.2571 (3)	0.0230 (8)	
H6EA	0.9299	0.1670	0.2123	0.028*	
C7E	0.9050 (3)	0.23280 (6)	0.2758 (3)	0.0217 (8)	
C8E	0.9334 (4)	0.13490 (7)	0.2961 (3)	0.0271 (9)	
H8EA	0.9950	0.1359	0.2701	0.033*	
H8EB	0.8759	0.1371	0.2484	0.033*	
C9E	0.9262 (4)	0.11393 (7)	0.3369 (3)	0.0298 (10)	
H9EA	0.8621	0.1128	0.3588	0.036*	
H9EB	0.9259	0.1040	0.2895	0.036*	
C10E	1.0104 (4)	0.10915 (7)	0.4123 (4)	0.0310 (10)	
H10E	1.0769	0.1100	0.4017	0.037*	
C11E	0.9950 (5)	0.10372 (9)	0.4936 (4)	0.0404 (13)	
H11I	0.9291	0.1027	0.5058	0.048*	
H11J	1.0500	0.1009	0.5386	0.048*	
O1F	0.1889 (3)	0.34007 (5)	0.3495 (2)	0.0281 (7)	
O2F	0.1725 (3)	0.30852 (5)	0.3170 (2)	0.0294 (7)	
O3F	0.1606 (3)	0.26731 (5)	0.6860 (2)	0.0278 (7)	
H3FB	0.1596	0.2552	0.6942	0.042*	
O4F	0.1696 (3)	0.25744 (5)	0.5453 (2)	0.0277 (7)	
N1F	0.1790 (3)	0.35313 (5)	0.5138 (2)	0.0204 (7)	
H1FA	0.1842	0.3567	0.4597	0.024*	
N2F	0.1788 (3)	0.32233 (6)	0.3722 (3)	0.0236 (8)	
C1F	0.1768 (3)	0.33346 (6)	0.5321 (3)	0.0184 (8)	
C2F	0.1754 (3)	0.31797 (7)	0.4668 (3)	0.0213 (8)	
C3F	0.1717 (3)	0.29781 (7)	0.4894 (3)	0.0222 (8)	
H3FA	0.1704	0.2881	0.4449	0.027*	
C4F	0.1699 (4)	0.29217 (7)	0.5777 (3)	0.0220 (8)	
C5F	0.1712 (3)	0.30720 (6)	0.6434 (3)	0.0214 (8)	
H5FA	0.1688	0.3035	0.7027	0.026*	
C6F	0.1758 (3)	0.32710 (6)	0.6230 (3)	0.0187 (8)	
H6FA	0.1782	0.3366	0.6686	0.022*	
C7F	0.1671 (3)	0.27088 (6)	0.6022 (3)	0.0203 (8)	
C8F	0.1731 (3)	0.36891 (6)	0.5800 (3)	0.0224 (8)	
H8FA	0.2385	0.3704	0.6188	0.027*	
H8FB	0.1232	0.3654	0.6175	0.027*	
C9F	0.1428 (3)	0.38856 (6)	0.5307 (3)	0.0206 (8)	
H9FA	0.0798	0.3866	0.4890	0.025*	
H9FB	0.1309	0.3986	0.5743	0.025*	
C10F	0.2228 (4)	0.39591 (7)	0.4798 (3)	0.0242 (9)	
H10F	0.2876	0.3980	0.5123	0.029*	
C11F	0.2083 (4)	0.39967 (7)	0.3924 (4)	0.0292 (10)	
H11K	0.1444	0.3978	0.3577	0.035*	
H11L	0.2619	0.4042	0.3654	0.035*	
O1G	0.4258 (3)	0.32758 (5)	0.5320 (2)	0.0301 (7)	
O2G	0.4339 (3)	0.35946 (5)	0.5614 (2)	0.0287 (7)	
O4G	0.4637 (2)	0.40970 (5)	0.3370 (2)	0.0236 (7)	
O3G	0.4661 (3)	0.39941 (5)	0.1950 (2)	0.0291 (7)	
H3GB	0.4708	0.4115	0.1868	0.044*	
N1G	0.4256 (3)	0.31428 (6)	0.3676 (3)	0.0261 (8)	
H1GA	0.4226	0.3109	0.4222	0.031*	
N2G	0.4334 (3)	0.34537 (6)	0.5087 (2)	0.0228 (7)	
C1G	0.4356 (3)	0.33379 (7)	0.3488 (3)	0.0221 (8)	
C2G	0.4396 (3)	0.34948 (6)	0.4150 (3)	0.0194 (8)	
C3G	0.4483 (3)	0.36957 (6)	0.3917 (3)	0.0189 (8)	
H3GA	0.4510	0.3795	0.4356	0.023*	
C4G	0.4528 (3)	0.37475 (7)	0.3034 (3)	0.0206 (8)	
C5G	0.4492 (3)	0.35973 (7)	0.2371 (3)	0.0223 (8)	
H5GA	0.4524	0.3633	0.1777	0.027*	
C6G	0.4412 (3)	0.34012 (7)	0.2590 (3)	0.0233 (8)	
H6GA	0.4392	0.3305	0.2140	0.028*	
C7G	0.4614 (3)	0.39604 (7)	0.2795 (3)	0.0220 (8)	
C8G	0.4195 (5)	0.29851 (8)	0.2980 (3)	0.0348 (11)	
H8GA	0.4837	0.2975	0.2762	0.042*	
H8GB	0.3669	0.3018	0.2473	0.042*	
C9G	0.3948 (6)	0.27852 (10)	0.3394 (5)	0.0552 (17)	
H9GA	0.3293	0.2796	0.3589	0.066*	
H9GB	0.3898	0.2682	0.2937	0.066*	
C10G	0.4694 (6)	0.27285 (10)	0.4144 (5)	0.0537 (17)	
H10G	0.5371	0.2724	0.4069	0.064*	
C11G	0.4460 (7)	0.26813 (10)	0.4959 (5)	0.0580 (19)	
H11M	0.3787	0.2685	0.5048	0.070*	
H11N	0.4971	0.2645	0.5431	0.070*	
O1H	0.6949 (2)	0.37269 (5)	0.1115 (2)	0.0233 (6)	
O2H	0.6687 (3)	0.34912 (5)	0.0112 (2)	0.0277 (7)	
O3H	0.6506 (3)	0.28037 (5)	0.0834 (2)	0.0298 (7)	
H3HB	0.6456	0.2683	0.0745	0.045*	
O4H	0.6609 (3)	0.27052 (5)	0.2283 (2)	0.0310 (8)	
N1H	0.7181 (3)	0.36459 (5)	0.2845 (3)	0.0197 (7)	
H1HA	0.7174	0.3739	0.2447	0.024*	
N2H	0.6824 (3)	0.35480 (5)	0.0906 (2)	0.0185 (7)	
C1H	0.7002 (3)	0.34570 (6)	0.2547 (3)	0.0170 (7)	
C2H	0.6824 (3)	0.34006 (6)	0.1616 (3)	0.0185 (7)	
C3H	0.6687 (3)	0.32022 (7)	0.1345 (3)	0.0216 (8)	
H3HA	0.6590	0.3170	0.0734	0.026*	
C4H	0.6694 (3)	0.30522 (6)	0.1979 (3)	0.0215 (8)	
C5H	0.6843 (3)	0.31012 (7)	0.2905 (3)	0.0223 (8)	
H5HA	0.6846	0.3000	0.3332	0.027*	
C6H	0.6984 (3)	0.32972 (6)	0.3180 (3)	0.0199 (8)	
H6HA	0.7071	0.3327	0.3794	0.024*	
C7H	0.6595 (4)	0.28398 (6)	0.1702 (3)	0.0247 (9)	
C8H	0.7385 (3)	0.37048 (7)	0.3792 (3)	0.0223 (8)	
H8HA	0.7837	0.3609	0.4138	0.027*	
H8HB	0.6756	0.3709	0.4035	0.027*	
C9H	0.7880 (3)	0.39128 (6)	0.3861 (3)	0.0200 (8)	
H9HA	0.8056	0.3950	0.4492	0.024*	
H9HB	0.8503	0.3908	0.3608	0.024*	
C10H	0.7186 (3)	0.40677 (6)	0.3371 (3)	0.0210 (8)	
H10H	0.6651	0.4113	0.3642	0.025*	
C11H	0.7283 (4)	0.41445 (8)	0.2579 (4)	0.0310 (10)	
H11O	0.7810	0.4103	0.2289	0.037*	
H11P	0.6824	0.4240	0.2311	0.037*	
O1I	0.9871 (3)	0.37631 (5)	0.2729 (2)	0.0249 (7)	
O2I	0.9665 (2)	0.35322 (5)	0.3704 (2)	0.0252 (7)	
O3I	0.9224 (3)	0.28461 (5)	0.2946 (2)	0.0305 (8)	
H3IB	0.9173	0.2726	0.3031	0.046*	
O4I	0.8981 (3)	0.27468 (5)	0.1495 (2)	0.0325 (8)	
N1I	0.9359 (3)	0.36902 (5)	0.0990 (2)	0.0185 (7)	
H1IA	0.9568	0.3781	0.1385	0.022*	
N2I	0.9692 (3)	0.35869 (5)	0.2916 (2)	0.0180 (7)	
C1I	0.9348 (3)	0.34994 (6)	0.1276 (3)	0.0187 (8)	
C2I	0.9492 (3)	0.34421 (6)	0.2196 (3)	0.0185 (8)	
C3I	0.9418 (3)	0.32425 (6)	0.2460 (3)	0.0193 (8)	
H3IA	0.9500	0.3210	0.3070	0.023*	
C4I	0.9222 (3)	0.30932 (6)	0.1815 (3)	0.0200 (8)	
C5I	0.9102 (3)	0.31459 (7)	0.0897 (3)	0.0238 (9)	
H5IA	0.8977	0.3046	0.0461	0.029*	
C6I	0.9163 (3)	0.33419 (7)	0.0627 (3)	0.0216 (8)	
H6IA	0.9084	0.3372	0.0015	0.026*	
C7I	0.9145 (4)	0.28810 (7)	0.2073 (3)	0.0242 (9)	
C8I	0.9044 (3)	0.37542 (7)	0.0059 (3)	0.0220 (8)	
H8IA	0.8448	0.3679	−0.0214	0.026*	
H8IB	0.9584	0.3728	−0.0285	0.026*	
C9I	0.8800 (3)	0.39791 (6)	0.0041 (3)	0.0222 (8)	
H9IA	0.8532	0.4019	−0.0573	0.027*	
H9IB	0.8280	0.4004	0.0408	0.027*	
C10I	0.9712 (3)	0.41012 (7)	0.0385 (3)	0.0235 (9)	
H10I	1.0231	0.4104	0.0043	0.028*	
C11I	0.9845 (4)	0.42081 (8)	0.1151 (4)	0.0301 (10)	
H11Q	0.9343	0.4209	0.1512	0.036*	
H11R	1.0440	0.4281	0.1321	0.036*	
O1J	0.6912 (2)	−0.04212 (5)	0.6067 (2)	0.0218 (6)	
O2J	0.7066 (2)	−0.01889 (5)	0.5094 (2)	0.0202 (6)	
O3J	0.7274 (3)	0.05019 (5)	0.5851 (2)	0.0235 (6)	
H3JB	0.7294	0.0623	0.5767	0.035*	
O4J	0.7450 (3)	0.05988 (5)	0.7296 (2)	0.0254 (7)	
N1J	0.7383 (3)	−0.03462 (5)	0.7804 (2)	0.0166 (6)	
H1JA	0.7226	−0.0438	0.7405	0.020*	
N2J	0.7042 (2)	−0.02428 (5)	0.5882 (2)	0.0153 (6)	
C1J	0.7335 (3)	−0.01560 (6)	0.7525 (2)	0.0115 (6)	
C2J	0.7178 (3)	−0.00976 (6)	0.6585 (2)	0.0129 (7)	
C3J	0.7194 (3)	0.01032 (6)	0.6330 (3)	0.0142 (7)	
H3JA	0.7106	0.0136	0.5721	0.017*	
C4J	0.7341 (3)	0.02532 (6)	0.6973 (3)	0.0156 (7)	
C5J	0.7469 (3)	0.02006 (6)	0.7898 (3)	0.0163 (7)	
H5JA	0.7555	0.0301	0.8334	0.020*	
C6J	0.7468 (3)	0.00046 (6)	0.8159 (3)	0.0160 (7)	
H6JA	0.7556	−0.0025	0.8772	0.019*	
C7J	0.7362 (3)	0.04660 (6)	0.6716 (3)	0.0166 (7)	
C8J	0.7683 (3)	−0.04080 (7)	0.8742 (3)	0.0208 (8)	
H8JA	0.7124	−0.0388	0.9070	0.025*	
H8JB	0.8256	−0.0328	0.9028	0.025*	
C9J	0.7975 (3)	−0.06297 (6)	0.8762 (3)	0.0208 (8)	
H9JA	0.8509	−0.0649	0.8405	0.025*	
H9JB	0.8241	−0.0668	0.9379	0.025*	
C10J	0.7098 (3)	−0.07612 (7)	0.8404 (3)	0.0247 (9)	
H10J	0.6582	−0.0771	0.8748	0.030*	
C11J	0.6981 (4)	−0.08646 (8)	0.7652 (4)	0.0336 (11)	
H11S	0.7476	−0.0860	0.7284	0.040*	
H11T	0.6403	−0.0943	0.7486	0.040*	
O1K	0.4841 (2)	0.53994 (5)	0.2662 (2)	0.0248 (7)	
O2K	0.4993 (3)	0.51635 (5)	0.3670 (2)	0.0246 (7)	
O3K	0.4947 (3)	0.44726 (5)	0.2956 (2)	0.0264 (7)	
H3KB	0.4958	0.4351	0.3047	0.040*	
O4K	0.4824 (3)	0.43708 (5)	0.1518 (3)	0.0308 (8)	
N1K	0.4536 (3)	0.53129 (5)	0.0940 (2)	0.0175 (7)	
H1KA	0.4580	0.5406	0.1340	0.021*	
N2K	0.4897 (2)	0.52186 (6)	0.2871 (2)	0.0169 (6)	
C1K	0.4668 (3)	0.51243 (6)	0.1231 (3)	0.0147 (7)	
C2K	0.4841 (3)	0.50701 (6)	0.2170 (3)	0.0156 (7)	
C3K	0.4928 (3)	0.48703 (6)	0.2444 (3)	0.0151 (7)	
H3KA	0.5037	0.4840	0.3057	0.018*	
C4K	0.4854 (3)	0.47175 (6)	0.1821 (3)	0.0167 (7)	
C5K	0.4724 (3)	0.47678 (6)	0.0893 (3)	0.0176 (7)	
H5KA	0.4694	0.4666	0.0466	0.021*	
C6K	0.4642 (3)	0.49629 (6)	0.0614 (2)	0.0158 (7)	
H6KA	0.4568	0.4991	−0.0001	0.019*	
C7K	0.4884 (3)	0.45065 (7)	0.2100 (3)	0.0191 (8)	
C8K	0.4319 (3)	0.53727 (6)	−0.0015 (3)	0.0183 (7)	
H8KA	0.3837	0.5280	−0.0351	0.022*	
H8KB	0.4939	0.5369	−0.0272	0.022*	
C9K	0.3878 (3)	0.55848 (7)	−0.0079 (3)	0.0213 (8)	
H9KA	0.3693	0.5622	−0.0710	0.026*	
H9KB	0.3266	0.5587	0.0190	0.026*	
C10K	0.4616 (3)	0.57339 (7)	0.0388 (3)	0.0264 (9)	
H10K	0.5154	0.5772	0.0103	0.032*	
C11K	0.4558 (4)	0.58158 (8)	0.1179 (4)	0.0353 (11)	
H11U	0.4030	0.5781	0.1482	0.042*	
H11V	0.5046	0.5908	0.1433	0.042*	
O1L	0.2305 (3)	−0.00317 (5)	−0.1522 (2)	0.0248 (7)	
O2L	0.2347 (3)	0.02862 (5)	−0.1806 (2)	0.0273 (7)	
O4L	0.2148 (2)	0.07949 (5)	0.0451 (2)	0.0256 (7)	
O3L	0.2203 (3)	0.06950 (5)	0.1883 (2)	0.0277 (7)	
H3LB	0.2187	0.0816	0.1966	0.042*	
N1L	0.2342 (3)	−0.01613 (5)	0.0147 (2)	0.0182 (7)	
H1LA	0.2352	−0.0198	−0.0401	0.022*	
N2L	0.2309 (3)	0.01460 (5)	−0.1266 (2)	0.0182 (7)	
C1L	0.2281 (3)	0.00347 (6)	0.0321 (3)	0.0161 (7)	
C2L	0.2269 (3)	0.01911 (6)	−0.0334 (3)	0.0166 (7)	
C3L	0.2234 (3)	0.03931 (6)	−0.0102 (3)	0.0177 (8)	
H3LA	0.2223	0.0491	−0.0543	0.021*	
C4L	0.2214 (3)	0.04480 (6)	0.0785 (3)	0.0160 (7)	
C5L	0.2211 (3)	0.02969 (6)	0.1438 (3)	0.0179 (8)	
H5LA	0.2185	0.0333	0.2032	0.021*	
C6L	0.2246 (3)	0.00993 (7)	0.1218 (3)	0.0188 (8)	
H6LA	0.2247	0.0004	0.1669	0.023*	
C7L	0.2189 (3)	0.06601 (6)	0.1037 (3)	0.0184 (7)	
C8L	0.2391 (3)	−0.03163 (7)	0.0840 (3)	0.0217 (8)	
H8LA	0.1769	−0.0315	0.1094	0.026*	
H8LB	0.2954	−0.0289	0.1322	0.026*	
C9L	0.2532 (4)	−0.05211 (7)	0.0439 (3)	0.0291 (10)	
H9LA	0.3177	−0.0523	0.0222	0.035*	
H9LB	0.2563	−0.0621	0.0912	0.035*	
C10L	0.1710 (4)	−0.05764 (8)	−0.0316 (4)	0.0318 (11)	
H10L	0.1049	−0.0579	−0.0197	0.038*	
C11L	0.1844 (5)	−0.06217 (9)	−0.1135 (4)	0.0395 (13)	
H11W	0.2495	−0.0621	−0.1281	0.047*	
H11X	0.1290	−0.0655	−0.1572	0.047*	

### Atomic displacement parameters (Å^2^)

**Table:**

	*U*^11^	*U*^22^	*U*^33^	*U*^12^	*U*^13^	*U*^23^
O1A	0.0284 (16)	0.0177 (14)	0.0163 (15)	0.0009 (11)	0.0062 (11)	0.0026 (11)
O2A	0.0267 (15)	0.0179 (14)	0.0146 (14)	0.0035 (11)	0.0031 (11)	−0.0001 (11)
O3A	0.0347 (17)	0.0170 (14)	0.0172 (14)	0.0002 (12)	0.0084 (12)	0.0030 (11)
O4A	0.0266 (15)	0.0151 (13)	0.0193 (14)	0.0011 (11)	0.0066 (11)	−0.0014 (11)
N1A	0.0199 (16)	0.0136 (15)	0.0124 (16)	0.0004 (12)	0.0019 (12)	0.0006 (11)
N2A	0.0163 (15)	0.0151 (15)	0.0104 (15)	0.0007 (11)	0.0028 (11)	−0.0020 (11)
C1A	0.0128 (16)	0.0147 (17)	0.0132 (17)	−0.0003 (12)	0.0030 (12)	−0.0016 (13)
C2A	0.0150 (16)	0.0120 (16)	0.0118 (17)	−0.0010 (12)	0.0040 (13)	−0.0002 (12)
C3A	0.0161 (17)	0.0129 (16)	0.0096 (16)	−0.0007 (12)	0.0026 (12)	−0.0022 (12)
C4A	0.0143 (16)	0.0120 (16)	0.0144 (18)	0.0007 (12)	0.0020 (13)	0.0020 (13)
C5A	0.0174 (17)	0.0153 (17)	0.0107 (17)	0.0007 (13)	0.0050 (13)	0.0023 (13)
C6A	0.0189 (18)	0.0142 (17)	0.0145 (19)	−0.0008 (13)	0.0044 (13)	−0.0018 (13)
C7A	0.0168 (17)	0.0184 (18)	0.0148 (18)	−0.0016 (13)	0.0059 (13)	0.0018 (13)
C8A	0.0208 (19)	0.0176 (18)	0.0134 (18)	−0.0004 (14)	0.0053 (14)	−0.0028 (13)
C9A	0.026 (2)	0.0182 (19)	0.024 (2)	0.0037 (15)	0.0068 (16)	0.0008 (15)
C10A	0.028 (2)	0.0145 (18)	0.027 (2)	−0.0028 (15)	0.0034 (17)	−0.0013 (15)
C11A	0.031 (2)	0.024 (2)	0.025 (2)	−0.0019 (17)	0.0085 (18)	0.0016 (17)
O1B	0.055 (2)	0.0258 (17)	0.0205 (18)	−0.0025 (15)	0.0039 (15)	0.0023 (13)
O2B	0.0396 (19)	0.0329 (18)	0.0142 (15)	0.0002 (14)	0.0034 (13)	−0.0018 (12)
O3B	0.042 (2)	0.0278 (17)	0.0241 (17)	0.0009 (15)	0.0092 (14)	−0.0020 (13)
O4B	0.052 (2)	0.0238 (16)	0.0250 (17)	0.0044 (15)	0.0064 (15)	0.0007 (13)
N1B	0.035 (2)	0.0255 (19)	0.0183 (19)	−0.0018 (15)	0.0013 (15)	−0.0035 (14)
N2B	0.0268 (19)	0.032 (2)	0.0174 (18)	0.0015 (15)	0.0005 (14)	0.0004 (15)
C1B	0.023 (2)	0.033 (2)	0.0122 (18)	−0.0021 (16)	0.0026 (14)	−0.0018 (15)
C2B	0.022 (2)	0.027 (2)	0.0162 (19)	−0.0031 (16)	0.0018 (15)	−0.0005 (15)
C3B	0.0191 (19)	0.029 (2)	0.0174 (19)	−0.0033 (16)	0.0013 (14)	−0.0022 (15)
C4B	0.020 (2)	0.029 (2)	0.024 (2)	−0.0029 (16)	0.0023 (16)	−0.0012 (16)
C5B	0.023 (2)	0.033 (2)	0.0162 (19)	−0.0031 (17)	0.0025 (15)	−0.0015 (16)
C6B	0.024 (2)	0.031 (2)	0.020 (2)	−0.0076 (17)	0.0024 (16)	−0.0025 (17)
C7B	0.021 (2)	0.028 (2)	0.025 (2)	−0.0013 (16)	0.0047 (16)	−0.0011 (16)
C8B	0.037 (3)	0.039 (3)	0.016 (2)	0.002 (2)	0.0010 (17)	−0.0082 (18)
C9B	0.036 (3)	0.043 (3)	0.026 (2)	−0.003 (2)	0.0057 (19)	−0.015 (2)
C10B	0.033 (2)	0.027 (2)	0.026 (2)	−0.0045 (18)	0.0051 (18)	−0.0105 (18)
C11B	0.039 (3)	0.049 (3)	0.039 (3)	−0.011 (2)	0.004 (2)	0.006 (2)
O1C	0.053 (2)	0.0191 (16)	0.0193 (17)	0.0006 (14)	0.0014 (14)	−0.0019 (12)
O2C	0.044 (2)	0.0255 (16)	0.0123 (15)	−0.0013 (14)	0.0026 (13)	−0.0019 (12)
O3C	0.0407 (19)	0.0181 (14)	0.0173 (15)	−0.0005 (13)	0.0051 (13)	0.0016 (11)
O4C	0.046 (2)	0.0169 (14)	0.0200 (15)	0.0008 (13)	0.0081 (14)	−0.0003 (11)
N1C	0.0298 (19)	0.0221 (18)	0.0131 (17)	0.0006 (14)	−0.0011 (13)	0.0014 (13)
N2C	0.0295 (19)	0.0201 (17)	0.0159 (17)	0.0000 (14)	0.0010 (13)	−0.0003 (13)
C1C	0.0185 (19)	0.0205 (19)	0.020 (2)	−0.0003 (14)	−0.0011 (14)	0.0010 (15)
C2C	0.0179 (18)	0.0214 (19)	0.0137 (18)	0.0005 (14)	−0.0010 (13)	−0.0028 (14)
C3C	0.0171 (18)	0.025 (2)	0.0118 (17)	0.0001 (14)	0.0007 (13)	−0.0007 (14)
C4C	0.0168 (18)	0.0185 (18)	0.0202 (19)	−0.0025 (14)	0.0040 (14)	−0.0005 (14)
C5C	0.0209 (19)	0.0200 (19)	0.0167 (19)	0.0013 (14)	0.0053 (14)	−0.0021 (14)
C6C	0.026 (2)	0.024 (2)	0.0113 (17)	0.0000 (16)	0.0019 (14)	−0.0012 (14)
C7C	0.0202 (19)	0.0203 (19)	0.0200 (19)	0.0004 (15)	0.0024 (15)	0.0006 (14)
C8C	0.036 (2)	0.021 (2)	0.0124 (19)	−0.0035 (17)	0.0027 (16)	0.0028 (14)
C9C	0.032 (2)	0.025 (2)	0.019 (2)	0.0000 (17)	0.0002 (16)	0.0059 (16)
C10C	0.030 (2)	0.018 (2)	0.029 (2)	0.0019 (16)	−0.0010 (17)	0.0039 (17)
C11C	0.043 (3)	0.035 (3)	0.037 (3)	0.003 (2)	0.000 (2)	−0.008 (2)
O1D	0.043 (2)	0.0209 (15)	0.0182 (16)	−0.0040 (13)	0.0069 (13)	0.0036 (12)
O2D	0.0356 (18)	0.0252 (16)	0.0180 (15)	−0.0035 (13)	0.0044 (12)	−0.0039 (12)
O3D	0.0329 (17)	0.0230 (15)	0.0166 (15)	0.0005 (13)	0.0042 (12)	0.0019 (11)
O4D	0.0269 (16)	0.0188 (14)	0.0176 (15)	0.0028 (11)	0.0037 (11)	−0.0007 (11)
N1D	0.038 (2)	0.0195 (17)	0.0149 (17)	−0.0031 (15)	0.0046 (14)	−0.0009 (13)
N2D	0.0230 (17)	0.0188 (16)	0.0157 (17)	−0.0042 (13)	0.0018 (13)	−0.0014 (12)
C1D	0.0194 (19)	0.0182 (18)	0.020 (2)	−0.0046 (14)	0.0049 (15)	−0.0013 (14)
C2D	0.0183 (18)	0.024 (2)	0.0127 (18)	−0.0010 (14)	0.0015 (14)	−0.0007 (14)
C3D	0.0123 (17)	0.0176 (18)	0.0194 (19)	−0.0016 (13)	0.0015 (13)	−0.0015 (14)
C4D	0.0155 (17)	0.0173 (18)	0.0197 (19)	−0.0005 (14)	0.0023 (14)	−0.0012 (14)
C5D	0.0152 (18)	0.025 (2)	0.018 (2)	−0.0033 (15)	−0.0004 (14)	−0.0007 (15)
C6D	0.0188 (18)	0.026 (2)	0.0140 (18)	−0.0043 (15)	0.0014 (14)	−0.0014 (15)
C7D	0.0141 (17)	0.0228 (19)	0.021 (2)	−0.0001 (14)	0.0054 (14)	−0.0026 (15)
C8D	0.049 (3)	0.019 (2)	0.021 (2)	−0.0059 (19)	0.0079 (19)	−0.0018 (16)
C9D	0.068 (4)	0.037 (3)	0.031 (3)	−0.005 (3)	0.015 (3)	−0.006 (2)
C10D	0.052 (3)	0.039 (3)	0.033 (3)	−0.018 (3)	0.004 (2)	−0.001 (2)
C11D	0.070 (4)	0.045 (3)	0.030 (3)	−0.009 (3)	0.013 (3)	0.005 (3)
O1E	0.044 (2)	0.0240 (17)	0.0203 (16)	−0.0058 (14)	0.0077 (14)	0.0004 (13)
O2E	0.048 (2)	0.0303 (18)	0.0170 (16)	−0.0088 (15)	0.0059 (14)	−0.0063 (13)
O3E	0.0410 (19)	0.0214 (16)	0.0210 (16)	0.0018 (13)	0.0032 (13)	0.0016 (12)
O4E	0.0409 (19)	0.0202 (15)	0.0239 (17)	0.0034 (13)	0.0109 (14)	−0.0020 (12)
N1E	0.0270 (19)	0.0189 (17)	0.0206 (18)	−0.0020 (14)	0.0017 (14)	−0.0031 (13)
N2E	0.0252 (18)	0.0263 (19)	0.0185 (18)	−0.0045 (14)	−0.0016 (14)	−0.0034 (14)
C1E	0.0182 (19)	0.022 (2)	0.020 (2)	−0.0044 (15)	0.0021 (14)	−0.0019 (15)
C2E	0.021 (2)	0.022 (2)	0.018 (2)	−0.0012 (15)	0.0021 (15)	−0.0004 (15)
C3E	0.022 (2)	0.029 (2)	0.015 (2)	−0.0044 (16)	0.0010 (15)	−0.0026 (16)
C4E	0.028 (2)	0.023 (2)	0.021 (2)	0.0012 (16)	0.0015 (16)	0.0004 (16)
C5E	0.034 (2)	0.022 (2)	0.0115 (19)	0.0008 (16)	0.0014 (15)	−0.0007 (15)
C6E	0.027 (2)	0.022 (2)	0.019 (2)	−0.0020 (16)	0.0026 (16)	−0.0011 (16)
C7E	0.029 (2)	0.021 (2)	0.0156 (19)	−0.0013 (16)	0.0022 (15)	0.0025 (15)
C8E	0.033 (2)	0.025 (2)	0.024 (2)	−0.0038 (18)	0.0051 (18)	−0.0059 (17)
C9E	0.036 (3)	0.024 (2)	0.031 (2)	−0.0097 (18)	0.0103 (19)	−0.0090 (17)
C10E	0.035 (3)	0.018 (2)	0.043 (3)	0.0016 (18)	0.014 (2)	0.0043 (18)
C11E	0.049 (3)	0.041 (3)	0.032 (3)	−0.005 (2)	0.008 (2)	0.000 (2)
O1F	0.042 (2)	0.0228 (16)	0.0207 (16)	−0.0007 (14)	0.0095 (14)	0.0061 (13)
O2F	0.044 (2)	0.0303 (18)	0.0144 (15)	−0.0058 (15)	0.0046 (13)	−0.0075 (12)
O3F	0.047 (2)	0.0190 (15)	0.0183 (16)	−0.0027 (13)	0.0084 (14)	0.0034 (12)
O4F	0.043 (2)	0.0189 (15)	0.0206 (16)	−0.0015 (13)	0.0049 (13)	−0.0017 (12)
N1F	0.0278 (18)	0.0176 (16)	0.0169 (17)	−0.0007 (13)	0.0067 (13)	0.0001 (12)
N2F	0.0234 (18)	0.029 (2)	0.0197 (19)	−0.0005 (14)	0.0062 (14)	0.0040 (14)
C1F	0.0192 (18)	0.0204 (19)	0.0152 (19)	0.0005 (14)	0.0019 (14)	0.0020 (14)
C2F	0.023 (2)	0.026 (2)	0.0142 (19)	0.0001 (16)	0.0025 (14)	0.0017 (15)
C3F	0.021 (2)	0.020 (2)	0.024 (2)	−0.0023 (15)	0.0030 (16)	0.0019 (16)
C4F	0.031 (2)	0.024 (2)	0.0113 (18)	−0.0021 (16)	0.0044 (15)	−0.0032 (15)
C5F	0.031 (2)	0.020 (2)	0.0122 (19)	−0.0016 (16)	0.0011 (15)	−0.0027 (14)
C6F	0.0215 (19)	0.0216 (19)	0.0126 (18)	0.0022 (15)	0.0021 (14)	0.0041 (14)
C7F	0.029 (2)	0.0159 (18)	0.0164 (19)	0.0030 (15)	0.0038 (15)	0.0019 (14)
C8F	0.028 (2)	0.021 (2)	0.0188 (19)	−0.0008 (16)	0.0078 (16)	0.0008 (15)
C9F	0.030 (2)	0.0165 (17)	0.0173 (18)	0.0013 (15)	0.0084 (15)	0.0018 (13)
C10F	0.028 (2)	0.024 (2)	0.021 (2)	−0.0046 (17)	0.0053 (16)	0.0016 (15)
C11F	0.031 (2)	0.028 (2)	0.031 (3)	−0.0020 (18)	0.0084 (19)	0.0032 (18)
O1G	0.040 (2)	0.0308 (18)	0.0200 (17)	−0.0060 (14)	0.0064 (14)	−0.0004 (13)
O2G	0.0388 (19)	0.0277 (16)	0.0194 (16)	−0.0006 (14)	0.0040 (13)	−0.0038 (12)
O4G	0.0254 (16)	0.0211 (15)	0.0253 (16)	−0.0008 (12)	0.0070 (12)	−0.0039 (12)
O3G	0.0375 (19)	0.0282 (17)	0.0236 (17)	−0.0035 (14)	0.0110 (14)	0.0039 (13)
N1G	0.035 (2)	0.0245 (18)	0.0182 (18)	−0.0031 (15)	0.0022 (15)	−0.0009 (14)
N2G	0.0249 (18)	0.0288 (19)	0.0137 (17)	−0.0020 (14)	0.0007 (13)	−0.0030 (13)
C1G	0.0180 (19)	0.025 (2)	0.022 (2)	0.0000 (15)	0.0000 (15)	−0.0020 (16)
C2G	0.0221 (19)	0.0218 (19)	0.0142 (18)	−0.0002 (15)	0.0034 (14)	0.0003 (14)
C3G	0.0174 (18)	0.024 (2)	0.0150 (18)	0.0011 (15)	0.0022 (14)	−0.0049 (14)
C4G	0.0157 (18)	0.024 (2)	0.022 (2)	0.0016 (15)	0.0026 (15)	0.0045 (15)
C5G	0.020 (2)	0.029 (2)	0.018 (2)	0.0043 (16)	0.0038 (15)	0.0023 (16)
C6G	0.024 (2)	0.028 (2)	0.018 (2)	0.0022 (17)	0.0047 (16)	−0.0037 (16)
C7G	0.0145 (17)	0.030 (2)	0.023 (2)	0.0005 (15)	0.0061 (15)	0.0000 (16)
C8G	0.053 (3)	0.028 (2)	0.024 (2)	−0.007 (2)	0.009 (2)	−0.0054 (18)
C9G	0.072 (5)	0.046 (3)	0.051 (4)	−0.023 (3)	0.018 (3)	−0.013 (3)
C10G	0.067 (5)	0.036 (3)	0.062 (4)	−0.013 (3)	0.023 (4)	−0.003 (3)
C11G	0.097 (6)	0.046 (4)	0.033 (3)	−0.019 (4)	0.015 (3)	0.001 (3)
O1H	0.0326 (17)	0.0159 (14)	0.0209 (16)	−0.0022 (12)	0.0025 (12)	−0.0001 (11)
O2H	0.042 (2)	0.0261 (16)	0.0149 (15)	0.0049 (14)	0.0041 (13)	−0.0010 (12)
O3H	0.048 (2)	0.0200 (16)	0.0207 (17)	−0.0012 (14)	0.0049 (14)	−0.0063 (12)
O4H	0.055 (2)	0.0154 (15)	0.0228 (17)	−0.0018 (14)	0.0068 (15)	0.0016 (12)
N1H	0.0261 (18)	0.0141 (15)	0.0184 (17)	0.0007 (13)	0.0023 (13)	−0.0007 (12)
N2H	0.0167 (16)	0.0203 (17)	0.0187 (17)	0.0030 (12)	0.0037 (12)	0.0041 (13)
C1H	0.0155 (17)	0.0198 (19)	0.0159 (18)	0.0025 (14)	0.0033 (14)	−0.0008 (14)
C2H	0.0178 (18)	0.0207 (19)	0.0168 (19)	0.0010 (14)	0.0025 (14)	0.0002 (14)
C3H	0.021 (2)	0.022 (2)	0.021 (2)	−0.0001 (15)	0.0034 (15)	−0.0037 (16)
C4H	0.026 (2)	0.0173 (19)	0.020 (2)	−0.0012 (15)	0.0024 (15)	−0.0024 (15)
C5H	0.026 (2)	0.024 (2)	0.017 (2)	−0.0019 (16)	0.0030 (15)	−0.0001 (15)
C6H	0.0206 (19)	0.0195 (19)	0.019 (2)	−0.0006 (15)	0.0022 (15)	−0.0011 (15)
C7H	0.036 (2)	0.0172 (19)	0.020 (2)	−0.0011 (17)	0.0038 (17)	−0.0026 (15)
C8H	0.026 (2)	0.024 (2)	0.017 (2)	−0.0011 (16)	0.0038 (15)	−0.0037 (15)
C9H	0.0169 (18)	0.0203 (19)	0.021 (2)	0.0015 (14)	−0.0010 (14)	−0.0058 (15)
C10H	0.0157 (18)	0.0174 (19)	0.029 (2)	−0.0017 (14)	0.0007 (15)	−0.0062 (16)
C11H	0.025 (2)	0.028 (2)	0.039 (3)	0.0005 (18)	0.0025 (19)	0.007 (2)
O1I	0.0305 (17)	0.0222 (16)	0.0217 (17)	−0.0039 (12)	0.0038 (13)	0.0016 (12)
O2I	0.0304 (17)	0.0309 (17)	0.0151 (15)	−0.0009 (13)	0.0061 (12)	0.0012 (12)
O3I	0.054 (2)	0.0197 (16)	0.0170 (16)	0.0025 (14)	0.0051 (14)	0.0039 (12)
O4I	0.053 (2)	0.0204 (16)	0.0222 (17)	0.0065 (14)	0.0012 (15)	0.0009 (12)
N1I	0.0218 (17)	0.0178 (16)	0.0155 (16)	0.0002 (13)	0.0022 (12)	0.0035 (12)
N2I	0.0187 (16)	0.0168 (16)	0.0181 (17)	0.0014 (12)	0.0021 (12)	0.0002 (12)
C1I	0.0170 (18)	0.0170 (18)	0.023 (2)	0.0007 (14)	0.0051 (15)	0.0016 (14)
C2I	0.0176 (18)	0.0194 (19)	0.019 (2)	0.0010 (14)	0.0048 (14)	−0.0023 (14)
C3I	0.0202 (19)	0.023 (2)	0.0147 (18)	0.0011 (15)	0.0039 (14)	0.0012 (15)
C4I	0.026 (2)	0.0194 (19)	0.0145 (18)	0.0011 (15)	0.0019 (14)	0.0019 (14)
C5I	0.031 (2)	0.0182 (19)	0.021 (2)	0.0058 (16)	0.0000 (16)	−0.0032 (15)
C6I	0.025 (2)	0.025 (2)	0.0143 (19)	0.0044 (16)	0.0005 (15)	−0.0008 (15)
C7I	0.032 (2)	0.021 (2)	0.020 (2)	0.0041 (17)	0.0032 (16)	0.0032 (15)
C8I	0.026 (2)	0.021 (2)	0.019 (2)	−0.0027 (16)	0.0043 (16)	0.0040 (15)
C9I	0.021 (2)	0.022 (2)	0.023 (2)	0.0034 (15)	0.0013 (15)	0.0056 (15)
C10I	0.022 (2)	0.025 (2)	0.024 (2)	0.0057 (16)	0.0068 (16)	0.0062 (16)
C11I	0.023 (2)	0.033 (2)	0.035 (3)	−0.0002 (18)	0.0035 (18)	0.000 (2)
O1J	0.0294 (16)	0.0182 (14)	0.0183 (15)	−0.0017 (12)	0.0059 (12)	−0.0008 (11)
O2J	0.0287 (16)	0.0217 (15)	0.0101 (14)	0.0009 (12)	0.0034 (11)	0.0001 (11)
O3J	0.0353 (17)	0.0175 (14)	0.0182 (15)	0.0012 (12)	0.0054 (12)	0.0020 (11)
O4J	0.0366 (18)	0.0218 (15)	0.0175 (15)	−0.0005 (13)	0.0038 (13)	−0.0030 (11)
N1J	0.0199 (16)	0.0186 (16)	0.0111 (15)	−0.0005 (12)	0.0022 (12)	0.0032 (12)
N2J	0.0119 (14)	0.0232 (17)	0.0109 (15)	−0.0011 (12)	0.0022 (11)	0.0001 (12)
C1J	0.0101 (15)	0.0185 (17)	0.0051 (15)	0.0022 (12)	−0.0007 (11)	0.0037 (12)
C2J	0.0161 (17)	0.0169 (17)	0.0056 (16)	−0.0008 (13)	0.0009 (12)	0.0009 (12)
C3J	0.0158 (17)	0.0147 (17)	0.0123 (18)	0.0004 (13)	0.0027 (13)	0.0023 (13)
C4J	0.0163 (17)	0.0175 (18)	0.0133 (18)	0.0027 (13)	0.0036 (13)	0.0024 (13)
C5J	0.0161 (17)	0.0239 (19)	0.0089 (17)	0.0025 (14)	0.0019 (13)	0.0016 (14)
C6J	0.0168 (17)	0.0196 (18)	0.0111 (17)	0.0003 (14)	0.0012 (13)	0.0017 (13)
C7J	0.0183 (18)	0.0155 (17)	0.0165 (19)	0.0030 (14)	0.0044 (14)	0.0030 (13)
C8J	0.025 (2)	0.025 (2)	0.0111 (18)	−0.0002 (16)	−0.0018 (14)	0.0051 (14)
C9J	0.0167 (18)	0.024 (2)	0.022 (2)	−0.0012 (14)	0.0038 (14)	0.0061 (15)
C10J	0.0171 (19)	0.031 (2)	0.026 (2)	0.0026 (16)	0.0030 (16)	0.0115 (17)
C11J	0.030 (2)	0.037 (3)	0.032 (3)	0.004 (2)	0.001 (2)	−0.003 (2)
O1K	0.0300 (17)	0.0266 (16)	0.0162 (15)	0.0047 (13)	−0.0005 (12)	0.0001 (12)
O2K	0.0327 (17)	0.0300 (17)	0.0114 (15)	−0.0044 (13)	0.0046 (12)	0.0016 (12)
O3K	0.0345 (18)	0.0232 (16)	0.0219 (17)	−0.0031 (13)	0.0055 (13)	0.0038 (12)
O4K	0.042 (2)	0.0253 (17)	0.0264 (18)	0.0028 (14)	0.0090 (15)	0.0015 (13)
N1K	0.0226 (17)	0.0216 (17)	0.0085 (15)	0.0026 (13)	0.0029 (12)	0.0007 (12)
N2K	0.0145 (15)	0.0263 (17)	0.0096 (15)	−0.0001 (12)	0.0015 (11)	0.0004 (12)
C1K	0.0101 (16)	0.0221 (18)	0.0120 (17)	0.0011 (13)	0.0024 (12)	−0.0007 (13)
C2K	0.0189 (18)	0.0204 (18)	0.0079 (16)	0.0012 (14)	0.0032 (13)	−0.0011 (13)
C3K	0.0130 (16)	0.0219 (19)	0.0107 (17)	0.0014 (13)	0.0029 (12)	−0.0014 (13)
C4K	0.0141 (17)	0.0233 (19)	0.0136 (18)	0.0034 (14)	0.0051 (13)	0.0024 (14)
C5K	0.0177 (18)	0.0218 (19)	0.0133 (18)	0.0044 (14)	0.0028 (13)	0.0005 (14)
C6K	0.0200 (18)	0.0230 (19)	0.0043 (15)	0.0044 (14)	0.0018 (12)	−0.0013 (13)
C7K	0.0149 (17)	0.027 (2)	0.0151 (18)	0.0032 (15)	0.0029 (13)	−0.0005 (15)
C8K	0.0227 (19)	0.0211 (19)	0.0104 (17)	−0.0020 (15)	0.0010 (14)	0.0002 (13)
C9K	0.0184 (18)	0.026 (2)	0.020 (2)	0.0017 (15)	0.0042 (14)	0.0042 (15)
C10K	0.020 (2)	0.030 (2)	0.028 (2)	−0.0015 (17)	0.0014 (17)	0.0022 (18)
C11K	0.034 (3)	0.037 (3)	0.032 (3)	0.009 (2)	−0.002 (2)	−0.009 (2)
O1L	0.0336 (17)	0.0234 (16)	0.0185 (16)	0.0028 (13)	0.0075 (13)	−0.0003 (12)
O2L	0.0363 (18)	0.0284 (17)	0.0184 (16)	−0.0001 (14)	0.0085 (13)	0.0079 (13)
O4L	0.0267 (16)	0.0218 (15)	0.0293 (18)	−0.0030 (12)	0.0075 (13)	0.0026 (12)
O3L	0.0333 (18)	0.0280 (17)	0.0223 (16)	0.0010 (13)	0.0066 (13)	−0.0019 (12)
N1L	0.0202 (17)	0.0203 (17)	0.0142 (16)	0.0037 (13)	0.0035 (12)	0.0041 (12)
N2L	0.0178 (16)	0.0224 (17)	0.0149 (17)	0.0008 (13)	0.0041 (12)	0.0007 (13)
C1L	0.0125 (16)	0.0208 (19)	0.0151 (18)	0.0011 (13)	0.0026 (13)	0.0040 (14)
C2L	0.0131 (17)	0.025 (2)	0.0119 (18)	0.0004 (14)	0.0028 (13)	0.0033 (14)
C3L	0.0146 (17)	0.0169 (18)	0.022 (2)	−0.0002 (13)	0.0054 (14)	0.0041 (14)
C4L	0.0115 (16)	0.0207 (18)	0.0164 (18)	0.0009 (13)	0.0044 (13)	0.0032 (14)
C5L	0.0124 (16)	0.024 (2)	0.0174 (19)	−0.0019 (14)	0.0027 (13)	0.0027 (15)
C6L	0.0191 (18)	0.024 (2)	0.0137 (19)	−0.0004 (15)	0.0029 (14)	0.0042 (15)
C7L	0.0131 (17)	0.0213 (19)	0.0216 (19)	0.0002 (14)	0.0054 (14)	0.0009 (15)
C8L	0.025 (2)	0.022 (2)	0.019 (2)	0.0035 (16)	0.0065 (15)	0.0071 (15)
C9L	0.035 (2)	0.023 (2)	0.032 (3)	0.0109 (18)	0.016 (2)	0.0112 (18)
C10L	0.029 (2)	0.026 (2)	0.042 (3)	0.0001 (18)	0.014 (2)	−0.001 (2)
C11L	0.042 (3)	0.031 (3)	0.045 (3)	0.004 (2)	0.009 (2)	−0.002 (2)

### Geometric parameters (Å, °)

**Table:**

O1A—N2A	1.249 (4)	O1G—N2G	1.247 (5)
O2A—N2A	1.234 (4)	O2G—N2G	1.229 (5)
O3A—C7A	1.311 (5)	O4G—C7G	1.253 (5)
O3A—H3AB	0.82	O3G—C7G	1.301 (5)
O4A—C7A	1.237 (5)	O3G—H3GB	0.82
N1A—C1A	1.342 (5)	N1G—C1G	1.344 (6)
N1A—C8A	1.457 (5)	N1G—C8G	1.477 (6)
N1A—H1AA	0.86	N1G—H1GA	0.86
N2A—C2A	1.439 (5)	N2G—C2G	1.450 (5)
C1A—C6A	1.410 (6)	C1G—C6G	1.427 (6)
C1A—C2A	1.446 (5)	C1G—C2G	1.439 (6)
C2A—C3A	1.393 (5)	C2G—C3G	1.396 (6)
C3A—C4A	1.382 (5)	C3G—C4G	1.380 (6)
C3A—H3AA	0.93	C3G—H3GA	0.93
C4A—C5A	1.409 (5)	C4G—C5G	1.409 (6)
C4A—C7A	1.481 (5)	C4G—C7G	1.476 (6)
C5A—C6A	1.374 (5)	C5G—C6G	1.359 (6)
C5A—H5AA	0.93	C5G—H5GA	0.93
C6A—H6AA	0.93	C6G—H6GA	0.93
C8A—C9A	1.534 (6)	C8G—C9G	1.532 (8)
C8A—H8AA	0.97	C8G—H8GA	0.97
C8A—H8AB	0.97	C8G—H8GB	0.97
C9A—C10A	1.472 (6)	C9G—C10G	1.424 (11)
C9A—H9AA	0.97	C9G—H9GA	0.97
C9A—H9AB	0.97	C9G—H9GB	0.97
C10A—C11A	1.322 (7)	C10G—C11G	1.350 (9)
C10A—H10A	0.93	C10G—H10G	0.93
C11A—H11A	0.93	C11G—H11M	0.93
C11A—H11B	0.93	C11G—H11N	0.93
O1B—N2B	1.240 (5)	O1H—N2H	1.239 (5)
O2B—N2B	1.225 (5)	O2H—N2H	1.235 (5)
O3B—C7B	1.310 (6)	O3H—C7H	1.312 (5)
O3B—H3BB	0.82	O3H—H3HB	0.82
O4B—C7B	1.244 (6)	O4H—C7H	1.251 (5)
N1B—C1B	1.343 (6)	N1H—C1H	1.347 (5)
N1B—C8B	1.460 (6)	N1H—C8H	1.456 (6)
N1B—H1BA	0.86	N1H—H1HA	0.86
N2B—C2B	1.440 (6)	N2H—C2H	1.452 (5)
C1B—C6B	1.411 (7)	C1H—C2H	1.429 (6)
C1B—C2B	1.431 (6)	C1H—C6H	1.432 (6)
C2B—C3B	1.382 (6)	C2H—C3H	1.389 (6)
C3B—C4B	1.385 (6)	C3H—C4H	1.381 (6)
C3B—H3BA	0.93	C3H—H3HA	0.93
C4B—C5B	1.411 (6)	C4H—C5H	1.409 (6)
C4B—C7B	1.480 (7)	C4H—C7H	1.478 (6)
C5B—C6B	1.374 (7)	C5H—C6H	1.376 (6)
C5B—H5BA	0.93	C5H—H5HA	0.93
C6B—H6BA	0.93	C6H—H6HA	0.93
C8B—C9B	1.504 (7)	C8H—C9H	1.534 (6)
C8B—H8BA	0.97	C8H—H8HA	0.97
C8B—H8BB	0.97	C8H—H8HB	0.97
C9B—C10B	1.511 (7)	C9H—C10H	1.497 (6)
C9B—H9BA	0.97	C9H—H9HA	0.97
C9B—H9BB	0.97	C9H—H9HB	0.97
C10B—C11B	1.307 (8)	C10H—C11H	1.321 (7)
C10B—H10B	0.93	C10H—H10H	0.93
C11B—H11C	0.93	C11H—H11O	0.93
C11B—H11D	0.93	C11H—H11P	0.93
O1C—N2C	1.249 (5)	O1I—N2I	1.242 (5)
O2C—N2C	1.228 (5)	O2I—N2I	1.245 (5)
O3C—C7C	1.322 (5)	O3I—C7I	1.319 (5)
O3C—H3CB	0.82	O3I—H3IB	0.82
O4C—C7C	1.236 (5)	O4I—C7I	1.242 (6)
N1C—C1C	1.350 (6)	N1I—C1I	1.345 (5)
N1C—C8C	1.470 (6)	N1I—C8I	1.455 (6)
N1C—H1CA	0.86	N1I—H1IA	0.86
N2C—C2C	1.459 (5)	N2I—C2I	1.442 (5)
C1C—C6C	1.416 (6)	C1I—C2I	1.416 (6)
C1C—C2C	1.429 (6)	C1I—C6I	1.427 (6)
C2C—C3C	1.385 (6)	C2I—C3I	1.399 (6)
C3C—C4C	1.386 (6)	C3I—C4I	1.384 (6)
C3C—H3CA	0.93	C3I—H3IA	0.93
C4C—C5C	1.414 (6)	C4I—C5I	1.406 (6)
C4C—C7C	1.464 (6)	C4I—C7I	1.477 (6)
C5C—C6C	1.364 (6)	C5I—C6I	1.377 (6)
C5C—H5CA	0.93	C5I—H5IA	0.93
C6C—H6CA	0.93	C6I—H6IA	0.93
C8C—C9C	1.529 (6)	C8I—C9I	1.536 (6)
C8C—H8CA	0.97	C8I—H8IA	0.97
C8C—H8CB	0.97	C8I—H8IB	0.97
C9C—C10C	1.501 (7)	C9I—C10I	1.484 (6)
C9C—H9CA	0.97	C9I—H9IA	0.97
C9C—H9CB	0.97	C9I—H9IB	0.97
C10C—C11C	1.320 (7)	C10I—C11I	1.340 (7)
C10C—H10C	0.93	C10I—H10I	0.93
C11C—H11E	0.93	C11I—H11Q	0.93
C11C—H11F	0.93	C11I—H11R	0.93
O1D—N2D	1.256 (5)	O1J—N2J	1.243 (5)
O2D—N2D	1.229 (5)	O2J—N2J	1.242 (4)
O3D—C7D	1.305 (5)	O3J—C7J	1.308 (5)
O3D—H3DB	0.82	O3J—H3JB	0.82
O4D—C7D	1.233 (5)	O4J—C7J	1.235 (5)
N1D—C1D	1.346 (5)	N1J—C1J	1.336 (5)
N1D—C8D	1.464 (6)	N1J—C8J	1.458 (5)
N1D—H1DA	0.86	N1J—H1JA	0.86
N2D—C2D	1.454 (5)	N2J—C2J	1.422 (5)
C1D—C2D	1.424 (6)	C1J—C6J	1.426 (6)
C1D—C6D	1.430 (6)	C1J—C2J	1.445 (5)
C2D—C3D	1.404 (6)	C2J—C3J	1.396 (5)
C3D—C4D	1.383 (6)	C3J—C4J	1.382 (6)
C3D—H3DA	0.93	C3J—H3JA	0.93
C4D—C5D	1.411 (6)	C4J—C5J	1.415 (5)
C4D—C7D	1.468 (6)	C4J—C7J	1.474 (5)
C5D—C6D	1.365 (6)	C5J—C6J	1.367 (6)
C5D—H5DA	0.93	C5J—H5JA	0.93
C6D—H6DA	0.93	C6J—H6JA	0.93
C8D—C9D	1.541 (8)	C8J—C9J	1.530 (6)
C8D—H8DA	0.97	C8J—H8JA	0.97
C8D—H8DB	0.97	C8J—H8JB	0.97
C9D—C10D	1.482 (8)	C9J—C10J	1.490 (6)
C9D—H9DA	0.97	C9J—H9JA	0.97
C9D—H9DB	0.97	C9J—H9JB	0.97
C10D—C11D	1.326 (8)	C10J—C11J	1.311 (7)
C10D—H10D	0.93	C10J—H10J	0.93
C11D—H11G	0.93	C11J—H11S	0.93
C11D—H11H	0.93	C11J—H11T	0.93
O1E—N2E	1.247 (5)	O1K—N2K	1.246 (5)
O2E—N2E	1.238 (5)	O2K—N2K	1.241 (5)
O3E—C7E	1.310 (5)	O3K—C7K	1.295 (5)
O3E—H3EB	0.82	O3K—H3KB	0.82
O4E—C7E	1.253 (5)	O4K—C7K	1.252 (5)
N1E—C1E	1.350 (6)	N1K—C1K	1.335 (5)
N1E—C8E	1.455 (6)	N1K—C8K	1.470 (5)
N1E—H1EA	0.86	N1K—H1KA	0.86
N2E—C2E	1.452 (6)	N2K—C2K	1.439 (5)
C1E—C6E	1.428 (6)	C1K—C6K	1.418 (5)
C1E—C2E	1.435 (6)	C1K—C2K	1.437 (5)
C2E—C3E	1.383 (6)	C2K—C3K	1.395 (6)
C3E—C4E	1.386 (6)	C3K—C4K	1.377 (6)
C3E—H3EA	0.93	C3K—H3KA	0.93
C4E—C5E	1.430 (6)	C4K—C5K	1.415 (6)
C4E—C7E	1.483 (6)	C4K—C7K	1.468 (6)
C5E—C6E	1.371 (6)	C5K—C6K	1.368 (6)
C5E—H5EA	0.93	C5K—H5KA	0.93
C6E—H6EA	0.93	C6K—H6KA	0.93
C8E—C9E	1.537 (7)	C8K—C9K	1.531 (6)
C8E—H8EA	0.97	C8K—H8KA	0.97
C8E—H8EB	0.97	C8K—H8KB	0.97
C9E—C10E	1.491 (8)	C9K—C10K	1.491 (6)
C9E—H9EA	0.97	C9K—H9KA	0.97
C9E—H9EB	0.97	C9K—H9KB	0.97
C10E—C11E	1.323 (8)	C10K—C11K	1.322 (7)
C10E—H10E	0.93	C10K—H10K	0.93
C11E—H11I	0.93	C11K—H11U	0.93
C11E—H11J	0.93	C11K—H11V	0.93
O1F—N2F	1.246 (5)	O1L—N2L	1.247 (5)
O2F—N2F	1.234 (5)	O2L—N2L	1.245 (5)
O3F—C7F	1.298 (5)	O4L—C7L	1.253 (5)
O3F—H3FB	0.82	O3L—C7L	1.289 (5)
O4F—C7F	1.244 (5)	O3L—H3LB	0.82
N1F—C1F	1.344 (5)	N1L—C1L	1.340 (5)
N1F—C8F	1.460 (5)	N1L—C8L	1.461 (5)
N1F—H1FA	0.86	N1L—H1LA	0.86
N2F—C2F	1.459 (6)	N2L—C2L	1.441 (5)
C1F—C2F	1.424 (6)	C1L—C6L	1.423 (6)
C1F—C6F	1.433 (6)	C1L—C2L	1.432 (6)
C2F—C3F	1.391 (6)	C2L—C3L	1.397 (6)
C3F—C4F	1.382 (6)	C3L—C4L	1.386 (6)
C3F—H3FA	0.93	C3L—H3LA	0.93
C4F—C5F	1.406 (6)	C4L—C5L	1.407 (6)
C4F—C7F	1.470 (6)	C4L—C7L	1.468 (6)
C5F—C6F	1.367 (6)	C5L—C6L	1.363 (6)
C5F—H5FA	0.93	C5L—H5LA	0.93
C6F—H6FA	0.93	C6L—H6LA	0.93
C8F—C9F	1.527 (6)	C8L—C9L	1.518 (7)
C8F—H8FA	0.97	C8L—H8LA	0.97
C8F—H8FB	0.97	C8L—H8LB	0.97
C9F—C10F	1.496 (6)	C9L—C10L	1.487 (8)
C9F—H9FA	0.97	C9L—H9LA	0.97
C9F—H9FB	0.97	C9L—H9LB	0.97
C10F—C11F	1.319 (7)	C10L—C11L	1.308 (8)
C10F—H10F	0.93	C10L—H10L	0.93
C11F—H11K	0.93	C11L—H11W	0.93
C11F—H11L	0.93	C11L—H11X	0.93
			
C7A—O3A—H3AB	109.5	C7G—O3G—H3GB	109.5
C1A—N1A—C8A	123.5 (3)	C1G—N1G—C8G	122.6 (4)
C1A—N1A—H1AA	118.2	C1G—N1G—H1GA	118.7
C8A—N1A—H1AA	118.2	C8G—N1G—H1GA	118.7
O2A—N2A—O1A	121.1 (3)	O2G—N2G—O1G	122.7 (4)
O2A—N2A—C2A	119.3 (3)	O2G—N2G—C2G	119.1 (4)
O1A—N2A—C2A	119.6 (3)	O1G—N2G—C2G	118.3 (4)
N1A—C1A—C6A	120.7 (4)	N1G—C1G—C6G	120.5 (4)
N1A—C1A—C2A	123.9 (4)	N1G—C1G—C2G	123.7 (4)
C6A—C1A—C2A	115.4 (3)	C6G—C1G—C2G	115.8 (4)
C3A—C2A—N2A	116.7 (3)	C3G—C2G—C1G	121.5 (4)
C3A—C2A—C1A	121.6 (4)	C3G—C2G—N2G	116.5 (4)
N2A—C2A—C1A	121.7 (3)	C1G—C2G—N2G	122.1 (4)
C4A—C3A—C2A	120.3 (3)	C4G—C3G—C2G	120.0 (4)
C4A—C3A—H3AA	119.9	C4G—C3G—H3GA	120.0
C2A—C3A—H3AA	119.9	C2G—C3G—H3GA	120.0
C3A—C4A—C5A	119.7 (3)	C3G—C4G—C5G	119.9 (4)
C3A—C4A—C7A	119.8 (3)	C3G—C4G—C7G	119.5 (4)
C5A—C4A—C7A	120.5 (3)	C5G—C4G—C7G	120.5 (4)
C6A—C5A—C4A	120.0 (4)	C6G—C5G—C4G	120.6 (4)
C6A—C5A—H5AA	120.0	C6G—C5G—H5GA	119.7
C4A—C5A—H5AA	120.0	C4G—C5G—H5GA	119.7
C5A—C6A—C1A	123.0 (4)	C5G—C6G—C1G	122.2 (4)
C5A—C6A—H6AA	118.5	C5G—C6G—H6GA	118.9
C1A—C6A—H6AA	118.5	C1G—C6G—H6GA	118.9
O4A—C7A—O3A	124.5 (4)	O4G—C7G—O3G	123.1 (4)
O4A—C7A—C4A	121.7 (4)	O4G—C7G—C4G	121.8 (4)
O3A—C7A—C4A	113.8 (3)	O3G—C7G—C4G	115.0 (4)
N1A—C8A—C9A	109.4 (3)	N1G—C8G—C9G	108.9 (4)
N1A—C8A—H8AA	109.8	N1G—C8G—H8GA	109.9
C9A—C8A—H8AA	109.8	C9G—C8G—H8GA	109.9
N1A—C8A—H8AB	109.8	N1G—C8G—H8GB	109.9
C9A—C8A—H8AB	109.8	C9G—C8G—H8GB	109.9
H8AA—C8A—H8AB	108.2	H8GA—C8G—H8GB	108.3
C10A—C9A—C8A	112.5 (4)	C10G—C9G—C8G	112.6 (6)
C10A—C9A—H9AA	109.1	C10G—C9G—H9GA	109.1
C8A—C9A—H9AA	109.1	C8G—C9G—H9GA	109.1
C10A—C9A—H9AB	109.1	C10G—C9G—H9GB	109.1
C8A—C9A—H9AB	109.1	C8G—C9G—H9GB	109.1
H9AA—C9A—H9AB	107.8	H9GA—C9G—H9GB	107.8
C11A—C10A—C9A	126.4 (5)	C11G—C10G—C9G	122.8 (8)
C11A—C10A—H10A	116.8	C11G—C10G—H10G	118.6
C9A—C10A—H10A	116.8	C9G—C10G—H10G	118.6
C10A—C11A—H11A	120.0	C10G—C11G—H11M	120.0
C10A—C11A—H11B	120.0	C10G—C11G—H11N	120.0
H11A—C11A—H11B	120.0	H11M—C11G—H11N	120.0
C7B—O3B—H3BB	109.5	C7H—O3H—H3HB	109.5
C1B—N1B—C8B	124.7 (4)	C1H—N1H—C8H	124.9 (4)
C1B—N1B—H1BA	117.7	C1H—N1H—H1HA	117.6
C8B—N1B—H1BA	117.7	C8H—N1H—H1HA	117.6
O2B—N2B—O1B	121.4 (4)	O2H—N2H—O1H	122.2 (4)
O2B—N2B—C2B	119.9 (4)	O2H—N2H—C2H	118.9 (4)
O1B—N2B—C2B	118.7 (4)	O1H—N2H—C2H	118.9 (4)
N1B—C1B—C6B	119.8 (4)	N1H—C1H—C2H	124.3 (4)
N1B—C1B—C2B	124.1 (4)	N1H—C1H—C6H	119.9 (4)
C6B—C1B—C2B	116.2 (4)	C2H—C1H—C6H	115.8 (4)
C3B—C2B—C1B	122.0 (4)	C3H—C2H—C1H	122.1 (4)
C3B—C2B—N2B	116.1 (4)	C3H—C2H—N2H	116.6 (4)
C1B—C2B—N2B	121.9 (4)	C1H—C2H—N2H	121.3 (4)
C2B—C3B—C4B	120.1 (4)	C4H—C3H—C2H	120.2 (4)
C2B—C3B—H3BA	119.9	C4H—C3H—H3HA	119.9
C4B—C3B—H3BA	119.9	C2H—C3H—H3HA	119.9
C3B—C4B—C5B	119.2 (4)	C3H—C4H—C5H	119.7 (4)
C3B—C4B—C7B	121.1 (4)	C3H—C4H—C7H	120.7 (4)
C5B—C4B—C7B	119.6 (4)	C5H—C4H—C7H	119.5 (4)
C6B—C5B—C4B	120.7 (4)	C6H—C5H—C4H	120.5 (4)
C6B—C5B—H5BA	119.7	C6H—C5H—H5HA	119.8
C4B—C5B—H5BA	119.7	C4H—C5H—H5HA	119.8
C5B—C6B—C1B	121.8 (4)	C5H—C6H—C1H	121.7 (4)
C5B—C6B—H6BA	119.1	C5H—C6H—H6HA	119.2
C1B—C6B—H6BA	119.1	C1H—C6H—H6HA	119.2
O4B—C7B—O3B	123.6 (4)	O4H—C7H—O3H	123.4 (4)
O4B—C7B—C4B	120.2 (4)	O4H—C7H—C4H	120.2 (4)
O3B—C7B—C4B	116.2 (4)	O3H—C7H—C4H	116.5 (4)
N1B—C8B—C9B	109.4 (4)	N1H—C8H—C9H	108.6 (4)
N1B—C8B—H8BA	109.8	N1H—C8H—H8HA	110.0
C9B—C8B—H8BA	109.8	C9H—C8H—H8HA	110.0
N1B—C8B—H8BB	109.8	N1H—C8H—H8HB	110.0
C9B—C8B—H8BB	109.8	C9H—C8H—H8HB	110.0
H8BA—C8B—H8BB	108.2	H8HA—C8H—H8HB	108.4
C8B—C9B—C10B	111.2 (4)	C10H—C9H—C8H	111.6 (3)
C8B—C9B—H9BA	109.4	C10H—C9H—H9HA	109.3
C10B—C9B—H9BA	109.4	C8H—C9H—H9HA	109.3
C8B—C9B—H9BB	109.4	C10H—C9H—H9HB	109.3
C10B—C9B—H9BB	109.4	C8H—C9H—H9HB	109.3
H9BA—C9B—H9BB	108.0	H9HA—C9H—H9HB	108.0
C11B—C10B—C9B	124.1 (5)	C11H—C10H—C9H	124.4 (4)
C11B—C10B—H10B	118.0	C11H—C10H—H10H	117.8
C9B—C10B—H10B	118.0	C9H—C10H—H10H	117.8
C10B—C11B—H11C	120.0	C10H—C11H—H11O	120.0
C10B—C11B—H11D	120.0	C10H—C11H—H11P	120.0
H11C—C11B—H11D	120.0	H11O—C11H—H11P	120.0
C7C—O3C—H3CB	109.5	C7I—O3I—H3IB	109.5
C1C—N1C—C8C	124.6 (4)	C1I—N1I—C8I	124.6 (4)
C1C—N1C—H1CA	117.7	C1I—N1I—H1IA	117.7
C8C—N1C—H1CA	117.7	C8I—N1I—H1IA	117.7
O2C—N2C—O1C	122.3 (4)	O1I—N2I—O2I	122.0 (4)
O2C—N2C—C2C	118.4 (4)	O1I—N2I—C2I	118.9 (4)
O1C—N2C—C2C	119.3 (4)	O2I—N2I—C2I	119.0 (4)
N1C—C1C—C6C	120.8 (4)	N1I—C1I—C2I	124.1 (4)
N1C—C1C—C2C	123.3 (4)	N1I—C1I—C6I	119.3 (4)
C6C—C1C—C2C	115.8 (4)	C2I—C1I—C6I	116.6 (4)
C3C—C2C—C1C	121.7 (4)	C3I—C2I—C1I	122.0 (4)
C3C—C2C—N2C	116.6 (4)	C3I—C2I—N2I	116.1 (4)
C1C—C2C—N2C	121.6 (4)	C1I—C2I—N2I	121.9 (4)
C2C—C3C—C4C	121.0 (4)	C4I—C3I—C2I	120.2 (4)
C2C—C3C—H3CA	119.5	C4I—C3I—H3IA	119.9
C4C—C3C—H3CA	119.5	C2I—C3I—H3IA	119.9
C3C—C4C—C5C	118.0 (4)	C3I—C4I—C5I	118.9 (4)
C3C—C4C—C7C	122.2 (4)	C3I—C4I—C7I	121.4 (4)
C5C—C4C—C7C	119.8 (4)	C5I—C4I—C7I	119.7 (4)
C6C—C5C—C4C	121.5 (4)	C6I—C5I—C4I	121.6 (4)
C6C—C5C—H5CA	119.3	C6I—C5I—H5IA	119.2
C4C—C5C—H5CA	119.3	C4I—C5I—H5IA	119.2
C5C—C6C—C1C	121.9 (4)	C5I—C6I—C1I	120.8 (4)
C5C—C6C—H6CA	119.0	C5I—C6I—H6IA	119.6
C1C—C6C—H6CA	119.0	C1I—C6I—H6IA	119.6
O4C—C7C—O3C	123.3 (4)	O4I—C7I—O3I	122.9 (4)
O4C—C7C—C4C	121.3 (4)	O4I—C7I—C4I	121.4 (4)
O3C—C7C—C4C	115.4 (4)	O3I—C7I—C4I	115.7 (4)
N1C—C8C—C9C	108.6 (4)	N1I—C8I—C9I	109.3 (4)
N1C—C8C—H8CA	110.0	N1I—C8I—H8IA	109.8
C9C—C8C—H8CA	110.0	C9I—C8I—H8IA	109.8
N1C—C8C—H8CB	110.0	N1I—C8I—H8IB	109.8
C9C—C8C—H8CB	110.0	C9I—C8I—H8IB	109.8
H8CA—C8C—H8CB	108.4	H8IA—C8I—H8IB	108.3
C10C—C9C—C8C	112.4 (4)	C10I—C9I—C8I	111.8 (4)
C10C—C9C—H9CA	109.1	C10I—C9I—H9IA	109.3
C8C—C9C—H9CA	109.1	C8I—C9I—H9IA	109.3
C10C—C9C—H9CB	109.1	C10I—C9I—H9IB	109.3
C8C—C9C—H9CB	109.1	C8I—C9I—H9IB	109.3
H9CA—C9C—H9CB	107.8	H9IA—C9I—H9IB	107.9
C11C—C10C—C9C	124.9 (5)	C11I—C10I—C9I	125.1 (4)
C11C—C10C—H10C	117.6	C11I—C10I—H10I	117.4
C9C—C10C—H10C	117.6	C9I—C10I—H10I	117.4
C10C—C11C—H11E	120.0	C10I—C11I—H11Q	120.0
C10C—C11C—H11F	120.0	C10I—C11I—H11R	120.0
H11E—C11C—H11F	120.0	H11Q—C11I—H11R	120.0
C7D—O3D—H3DB	109.5	C7J—O3J—H3JB	109.5
C1D—N1D—C8D	124.5 (4)	C1J—N1J—C8J	124.3 (4)
C1D—N1D—H1DA	117.8	C1J—N1J—H1JA	117.8
C8D—N1D—H1DA	117.8	C8J—N1J—H1JA	117.8
O2D—N2D—O1D	123.1 (4)	O2J—N2J—O1J	121.2 (3)
O2D—N2D—C2D	119.2 (4)	O2J—N2J—C2J	119.3 (3)
O1D—N2D—C2D	117.7 (3)	O1J—N2J—C2J	119.5 (3)
N1D—C1D—C2D	124.9 (4)	N1J—C1J—C6J	120.7 (3)
N1D—C1D—C6D	119.2 (4)	N1J—C1J—C2J	123.7 (4)
C2D—C1D—C6D	115.9 (4)	C6J—C1J—C2J	115.6 (3)
C3D—C2D—C1D	122.3 (4)	C3J—C2J—N2J	117.2 (3)
C3D—C2D—N2D	115.5 (4)	C3J—C2J—C1J	121.4 (4)
C1D—C2D—N2D	122.1 (4)	N2J—C2J—C1J	121.4 (3)
C4D—C3D—C2D	119.8 (4)	C4J—C3J—C2J	120.8 (4)
C4D—C3D—H3DA	120.1	C4J—C3J—H3JA	119.6
C2D—C3D—H3DA	120.1	C2J—C3J—H3JA	119.6
C3D—C4D—C5D	118.8 (4)	C3J—C4J—C5J	119.0 (4)
C3D—C4D—C7D	119.9 (4)	C3J—C4J—C7J	121.5 (4)
C5D—C4D—C7D	121.2 (4)	C5J—C4J—C7J	119.4 (4)
C6D—C5D—C4D	122.0 (4)	C6J—C5J—C4J	120.9 (4)
C6D—C5D—H5DA	119.0	C6J—C5J—H5JA	119.5
C4D—C5D—H5DA	119.0	C4J—C5J—H5JA	119.5
C5D—C6D—C1D	121.1 (4)	C5J—C6J—C1J	122.3 (4)
C5D—C6D—H6DA	119.4	C5J—C6J—H6JA	118.9
C1D—C6D—H6DA	119.4	C1J—C6J—H6JA	118.9
O4D—C7D—O3D	123.5 (4)	O4J—C7J—O3J	123.5 (4)
O4D—C7D—C4D	122.1 (4)	O4J—C7J—C4J	120.7 (4)
O3D—C7D—C4D	114.3 (4)	O3J—C7J—C4J	115.7 (4)
N1D—C8D—C9D	108.9 (4)	N1J—C8J—C9J	108.7 (4)
N1D—C8D—H8DA	109.9	N1J—C8J—H8JA	109.9
C9D—C8D—H8DA	109.9	C9J—C8J—H8JA	109.9
N1D—C8D—H8DB	109.9	N1J—C8J—H8JB	109.9
C9D—C8D—H8DB	109.9	C9J—C8J—H8JB	109.9
H8DA—C8D—H8DB	108.3	H8JA—C8J—H8JB	108.3
C10D—C9D—C8D	110.1 (5)	C10J—C9J—C8J	112.4 (4)
C10D—C9D—H9DA	109.6	C10J—C9J—H9JA	109.1
C8D—C9D—H9DA	109.6	C8J—C9J—H9JA	109.1
C10D—C9D—H9DB	109.6	C10J—C9J—H9JB	109.1
C8D—C9D—H9DB	109.6	C8J—C9J—H9JB	109.1
H9DA—C9D—H9DB	108.2	H9JA—C9J—H9JB	107.9
C11D—C10D—C9D	123.8 (6)	C11J—C10J—C9J	126.5 (4)
C11D—C10D—H10D	118.1	C11J—C10J—H10J	116.8
C9D—C10D—H10D	118.1	C9J—C10J—H10J	116.8
C10D—C11D—H11G	120.0	C10J—C11J—H11S	120.0
C10D—C11D—H11H	120.0	C10J—C11J—H11T	120.0
H11G—C11D—H11H	120.0	H11S—C11J—H11T	120.0
C7E—O3E—H3EB	109.5	C7K—O3K—H3KB	109.5
C1E—N1E—C8E	122.8 (4)	C1K—N1K—C8K	124.7 (4)
C1E—N1E—H1EA	118.6	C1K—N1K—H1KA	117.6
C8E—N1E—H1EA	118.6	C8K—N1K—H1KA	117.6
O2E—N2E—O1E	122.2 (4)	O2K—N2K—O1K	121.5 (4)
O2E—N2E—C2E	118.8 (4)	O2K—N2K—C2K	119.2 (4)
O1E—N2E—C2E	119.0 (4)	O1K—N2K—C2K	119.3 (3)
N1E—C1E—C6E	119.3 (4)	N1K—C1K—C6K	121.0 (4)
N1E—C1E—C2E	125.0 (4)	N1K—C1K—C2K	123.3 (4)
C6E—C1E—C2E	115.8 (4)	C6K—C1K—C2K	115.7 (4)
C3E—C2E—C1E	122.0 (4)	C3K—C2K—C1K	121.5 (4)
C3E—C2E—N2E	116.9 (4)	C3K—C2K—N2K	116.8 (3)
C1E—C2E—N2E	121.1 (4)	C1K—C2K—N2K	121.6 (4)
C2E—C3E—C4E	121.0 (4)	C4K—C3K—C2K	121.0 (4)
C2E—C3E—H3EA	119.5	C4K—C3K—H3KA	119.5
C4E—C3E—H3EA	119.5	C2K—C3K—H3KA	119.5
C3E—C4E—C5E	118.6 (4)	C3K—C4K—C5K	118.4 (4)
C3E—C4E—C7E	121.0 (4)	C3K—C4K—C7K	121.5 (4)
C5E—C4E—C7E	120.4 (4)	C5K—C4K—C7K	120.1 (4)
C6E—C5E—C4E	120.6 (4)	C6K—C5K—C4K	121.3 (4)
C6E—C5E—H5EA	119.7	C6K—C5K—H5KA	119.4
C4E—C5E—H5EA	119.7	C4K—C5K—H5KA	119.4
C5E—C6E—C1E	122.1 (4)	C5K—C6K—C1K	122.0 (4)
C5E—C6E—H6EA	119.0	C5K—C6K—H6KA	119.0
C1E—C6E—H6EA	119.0	C1K—C6K—H6KA	119.0
O4E—C7E—O3E	122.8 (4)	O4K—C7K—O3K	123.6 (4)
O4E—C7E—C4E	122.1 (4)	O4K—C7K—C4K	120.0 (4)
O3E—C7E—C4E	115.1 (4)	O3K—C7K—C4K	116.4 (4)
N1E—C8E—C9E	109.8 (4)	N1K—C8K—C9K	108.9 (3)
N1E—C8E—H8EA	109.7	N1K—C8K—H8KA	109.9
C9E—C8E—H8EA	109.7	C9K—C8K—H8KA	109.9
N1E—C8E—H8EB	109.7	N1K—C8K—H8KB	109.9
C9E—C8E—H8EB	109.7	C9K—C8K—H8KB	109.9
H8EA—C8E—H8EB	108.2	H8KA—C8K—H8KB	108.3
C10E—C9E—C8E	114.1 (4)	C10K—C9K—C8K	111.8 (4)
C10E—C9E—H9EA	108.7	C10K—C9K—H9KA	109.3
C8E—C9E—H9EA	108.7	C8K—C9K—H9KA	109.3
C10E—C9E—H9EB	108.7	C10K—C9K—H9KB	109.3
C8E—C9E—H9EB	108.7	C8K—C9K—H9KB	109.3
H9EA—C9E—H9EB	107.6	H9KA—C9K—H9KB	107.9
C11E—C10E—C9E	123.1 (5)	C11K—C10K—C9K	124.5 (5)
C11E—C10E—H10E	118.4	C11K—C10K—H10K	117.8
C9E—C10E—H10E	118.4	C9K—C10K—H10K	117.8
C10E—C11E—H11I	120.0	C10K—C11K—H11U	120.0
C10E—C11E—H11J	120.0	C10K—C11K—H11V	120.0
H11I—C11E—H11J	120.0	H11U—C11K—H11V	120.0
C7F—O3F—H3FB	109.5	C7L—O3L—H3LB	109.5
C1F—N1F—C8F	124.2 (4)	C1L—N1L—C8L	123.4 (4)
C1F—N1F—H1FA	117.9	C1L—N1L—H1LA	118.3
C8F—N1F—H1FA	117.9	C8L—N1L—H1LA	118.3
O2F—N2F—O1F	121.7 (4)	O1L—N2L—O2L	120.9 (4)
O2F—N2F—C2F	119.7 (4)	O1L—N2L—C2L	119.9 (4)
O1F—N2F—C2F	118.6 (4)	O2L—N2L—C2L	119.2 (4)
N1F—C1F—C2F	124.6 (4)	N1L—C1L—C6L	119.6 (4)
N1F—C1F—C6F	119.3 (4)	N1L—C1L—C2L	124.9 (4)
C2F—C1F—C6F	116.1 (4)	C6L—C1L—C2L	115.5 (4)
C3F—C2F—C1F	122.1 (4)	C3L—C2L—C1L	122.0 (4)
C3F—C2F—N2F	116.1 (4)	C3L—C2L—N2L	116.9 (4)
C1F—C2F—N2F	121.8 (4)	C1L—C2L—N2L	121.1 (4)
C4F—C3F—C2F	120.3 (4)	C4L—C3L—C2L	120.2 (4)
C4F—C3F—H3FA	119.8	C4L—C3L—H3LA	119.9
C2F—C3F—H3FA	119.8	C2L—C3L—H3LA	119.9
C3F—C4F—C5F	118.6 (4)	C3L—C4L—C5L	118.8 (4)
C3F—C4F—C7F	120.6 (4)	C3L—C4L—C7L	120.5 (4)
C5F—C4F—C7F	120.8 (4)	C5L—C4L—C7L	120.7 (4)
C6F—C5F—C4F	122.2 (4)	C6L—C5L—C4L	121.4 (4)
C6F—C5F—H5FA	118.9	C6L—C5L—H5LA	119.3
C4F—C5F—H5FA	118.9	C4L—C5L—H5LA	119.3
C5F—C6F—C1F	120.6 (4)	C5L—C6L—C1L	122.0 (4)
C5F—C6F—H6FA	119.7	C5L—C6L—H6LA	119.0
C1F—C6F—H6FA	119.7	C1L—C6L—H6LA	119.0
O4F—C7F—O3F	123.2 (4)	O4L—C7L—O3L	123.6 (4)
O4F—C7F—C4F	121.4 (4)	O4L—C7L—C4L	120.8 (4)
O3F—C7F—C4F	115.4 (4)	O3L—C7L—C4L	115.5 (4)
N1F—C8F—C9F	109.3 (3)	N1L—C8L—C9L	110.4 (4)
N1F—C8F—H8FA	109.8	N1L—C8L—H8LA	109.6
C9F—C8F—H8FA	109.8	C9L—C8L—H8LA	109.6
N1F—C8F—H8FB	109.8	N1L—C8L—H8LB	109.6
C9F—C8F—H8FB	109.8	C9L—C8L—H8LB	109.6
H8FA—C8F—H8FB	108.3	H8LA—C8L—H8LB	108.1
C10F—C9F—C8F	112.1 (4)	C10L—C9L—C8L	113.7 (4)
C10F—C9F—H9FA	109.2	C10L—C9L—H9LA	108.8
C8F—C9F—H9FA	109.2	C8L—C9L—H9LA	108.8
C10F—C9F—H9FB	109.2	C10L—C9L—H9LB	108.8
C8F—C9F—H9FB	109.2	C8L—C9L—H9LB	108.8
H9FA—C9F—H9FB	107.9	H9LA—C9L—H9LB	107.7
C11F—C10F—C9F	125.0 (4)	C11L—C10L—C9L	125.1 (5)
C11F—C10F—H10F	117.5	C11L—C10L—H10L	117.4
C9F—C10F—H10F	117.5	C9L—C10L—H10L	117.4
C10F—C11F—H11K	120.0	C10L—C11L—H11W	120.0
C10F—C11F—H11L	120.0	C10L—C11L—H11X	120.0
H11K—C11F—H11L	120.0	H11W—C11L—H11X	120.0
			
C8A—N1A—C1A—C6A	2.2 (6)	C8G—N1G—C1G—C6G	−0.2 (7)
C8A—N1A—C1A—C2A	−176.0 (4)	C8G—N1G—C1G—C2G	178.6 (4)
O2A—N2A—C2A—C3A	−6.8 (5)	N1G—C1G—C2G—C3G	−178.8 (4)
O1A—N2A—C2A—C3A	174.4 (3)	C6G—C1G—C2G—C3G	0.0 (6)
O2A—N2A—C2A—C1A	175.6 (3)	N1G—C1G—C2G—N2G	0.3 (7)
O1A—N2A—C2A—C1A	−3.2 (5)	C6G—C1G—C2G—N2G	179.1 (4)
N1A—C1A—C2A—C3A	179.7 (4)	O2G—N2G—C2G—C3G	1.5 (6)
C6A—C1A—C2A—C3A	1.4 (5)	O1G—N2G—C2G—C3G	−179.9 (4)
N1A—C1A—C2A—N2A	−2.9 (6)	O2G—N2G—C2G—C1G	−177.7 (4)
C6A—C1A—C2A—N2A	178.8 (3)	O1G—N2G—C2G—C1G	1.0 (6)
N2A—C2A—C3A—C4A	−178.7 (3)	C1G—C2G—C3G—C4G	0.3 (6)
C1A—C2A—C3A—C4A	−1.2 (6)	N2G—C2G—C3G—C4G	−178.9 (4)
C2A—C3A—C4A—C5A	1.0 (6)	C2G—C3G—C4G—C5G	−0.4 (6)
C2A—C3A—C4A—C7A	179.7 (3)	C2G—C3G—C4G—C7G	179.7 (4)
C3A—C4A—C5A—C6A	−1.1 (6)	C3G—C4G—C5G—C6G	0.1 (6)
C7A—C4A—C5A—C6A	−179.8 (4)	C7G—C4G—C5G—C6G	−180.0 (4)
C4A—C5A—C6A—C1A	1.4 (6)	C4G—C5G—C6G—C1G	0.2 (7)
N1A—C1A—C6A—C5A	−179.9 (4)	N1G—C1G—C6G—C5G	178.6 (4)
C2A—C1A—C6A—C5A	−1.5 (6)	C2G—C1G—C6G—C5G	−0.3 (6)
C3A—C4A—C7A—O4A	−0.7 (6)	C3G—C4G—C7G—O4G	−0.8 (6)
C5A—C4A—C7A—O4A	178.0 (4)	C5G—C4G—C7G—O4G	179.3 (4)
C3A—C4A—C7A—O3A	179.1 (3)	C3G—C4G—C7G—O3G	179.4 (4)
C5A—C4A—C7A—O3A	−2.2 (5)	C5G—C4G—C7G—O3G	−0.6 (6)
C1A—N1A—C8A—C9A	165.6 (4)	C1G—N1G—C8G—C9G	−172.8 (5)
N1A—C8A—C9A—C10A	65.8 (5)	N1G—C8G—C9G—C10G	−59.6 (7)
C8A—C9A—C10A—C11A	−121.7 (5)	C8G—C9G—C10G—C11G	126.6 (7)
C8B—N1B—C1B—C6B	7.5 (7)	C8H—N1H—C1H—C2H	−178.6 (4)
C8B—N1B—C1B—C2B	−171.8 (4)	C8H—N1H—C1H—C6H	1.3 (6)
N1B—C1B—C2B—C3B	176.1 (4)	N1H—C1H—C2H—C3H	177.2 (4)
C6B—C1B—C2B—C3B	−3.2 (6)	C6H—C1H—C2H—C3H	−2.7 (6)
N1B—C1B—C2B—N2B	−0.9 (7)	N1H—C1H—C2H—N2H	0.0 (6)
C6B—C1B—C2B—N2B	179.8 (4)	C6H—C1H—C2H—N2H	−179.9 (3)
O2B—N2B—C2B—C3B	−4.7 (6)	O2H—N2H—C2H—C3H	1.1 (6)
O1B—N2B—C2B—C3B	176.2 (4)	O1H—N2H—C2H—C3H	−179.6 (4)
O2B—N2B—C2B—C1B	172.5 (4)	O2H—N2H—C2H—C1H	178.5 (4)
O1B—N2B—C2B—C1B	−6.6 (6)	O1H—N2H—C2H—C1H	−2.3 (6)
C1B—C2B—C3B—C4B	2.4 (7)	C1H—C2H—C3H—C4H	1.7 (6)
N2B—C2B—C3B—C4B	179.6 (4)	N2H—C2H—C3H—C4H	179.0 (4)
C2B—C3B—C4B—C5B	−0.8 (6)	C2H—C3H—C4H—C5H	−0.1 (7)
C2B—C3B—C4B—C7B	−177.6 (4)	C2H—C3H—C4H—C7H	−177.2 (4)
C3B—C4B—C5B—C6B	0.2 (7)	C3H—C4H—C5H—C6H	−0.2 (7)
C7B—C4B—C5B—C6B	177.0 (4)	C7H—C4H—C5H—C6H	176.9 (4)
C4B—C5B—C6B—C1B	−1.0 (7)	C4H—C5H—C6H—C1H	−1.0 (7)
N1B—C1B—C6B—C5B	−176.8 (4)	N1H—C1H—C6H—C5H	−177.5 (4)
C2B—C1B—C6B—C5B	2.5 (6)	C2H—C1H—C6H—C5H	2.4 (6)
C3B—C4B—C7B—O4B	−178.1 (4)	C3H—C4H—C7H—O4H	179.6 (4)
C5B—C4B—C7B—O4B	5.1 (7)	C5H—C4H—C7H—O4H	2.5 (7)
C3B—C4B—C7B—O3B	1.6 (6)	C3H—C4H—C7H—O3H	0.3 (7)
C5B—C4B—C7B—O3B	−175.2 (4)	C5H—C4H—C7H—O3H	−176.8 (4)
C1B—N1B—C8B—C9B	160.1 (4)	C1H—N1H—C8H—C9H	161.8 (4)
N1B—C8B—C9B—C10B	63.9 (5)	N1H—C8H—C9H—C10H	62.3 (5)
C8B—C9B—C10B—C11B	−109.4 (6)	C8H—C9H—C10H—C11H	−104.2 (5)
C8C—N1C—C1C—C6C	2.9 (7)	C8I—N1I—C1I—C2I	−169.0 (4)
C8C—N1C—C1C—C2C	−175.9 (4)	C8I—N1I—C1I—C6I	9.8 (6)
N1C—C1C—C2C—C3C	176.2 (4)	N1I—C1I—C2I—C3I	176.4 (4)
C6C—C1C—C2C—C3C	−2.7 (6)	C6I—C1I—C2I—C3I	−2.5 (6)
N1C—C1C—C2C—N2C	−2.3 (6)	N1I—C1I—C2I—N2I	−1.6 (6)
C6C—C1C—C2C—N2C	178.8 (4)	C6I—C1I—C2I—N2I	179.5 (4)
O2C—N2C—C2C—C3C	−4.3 (6)	O1I—N2I—C2I—C3I	172.6 (4)
O1C—N2C—C2C—C3C	175.7 (4)	O2I—N2I—C2I—C3I	−8.5 (5)
O2C—N2C—C2C—C1C	174.3 (4)	O1I—N2I—C2I—C1I	−9.3 (6)
O1C—N2C—C2C—C1C	−5.7 (6)	O2I—N2I—C2I—C1I	169.6 (4)
C1C—C2C—C3C—C4C	1.5 (6)	C1I—C2I—C3I—C4I	1.6 (6)
N2C—C2C—C3C—C4C	−179.9 (4)	N2I—C2I—C3I—C4I	179.7 (4)
C2C—C3C—C4C—C5C	0.5 (6)	C2I—C3I—C4I—C5I	0.1 (6)
C2C—C3C—C4C—C7C	−179.5 (4)	C2I—C3I—C4I—C7I	179.6 (4)
C3C—C4C—C5C—C6C	−1.2 (6)	C3I—C4I—C5I—C6I	−0.7 (7)
C7C—C4C—C5C—C6C	178.8 (4)	C7I—C4I—C5I—C6I	179.7 (4)
C4C—C5C—C6C—C1C	−0.1 (7)	C4I—C5I—C6I—C1I	−0.3 (7)
N1C—C1C—C6C—C5C	−177.0 (4)	N1I—C1I—C6I—C5I	−177.1 (4)
C2C—C1C—C6C—C5C	2.0 (6)	C2I—C1I—C6I—C5I	1.8 (6)
C3C—C4C—C7C—O4C	179.6 (4)	C3I—C4I—C7I—O4I	−179.1 (4)
C5C—C4C—C7C—O4C	−0.3 (6)	C5I—C4I—C7I—O4I	0.5 (7)
C3C—C4C—C7C—O3C	1.4 (6)	C3I—C4I—C7I—O3I	3.3 (6)
C5C—C4C—C7C—O3C	−178.6 (4)	C5I—C4I—C7I—O3I	−177.1 (4)
C1C—N1C—C8C—C9C	162.3 (4)	C1I—N1I—C8I—C9I	160.0 (4)
N1C—C8C—C9C—C10C	64.6 (5)	N1I—C8I—C9I—C10I	64.0 (5)
C8C—C9C—C10C—C11C	−107.0 (6)	C8I—C9I—C10I—C11I	−112.9 (5)
C8D—N1D—C1D—C2D	178.9 (4)	C8J—N1J—C1J—C6J	−7.4 (6)
C8D—N1D—C1D—C6D	0.0 (7)	C8J—N1J—C1J—C2J	170.8 (4)
N1D—C1D—C2D—C3D	−179.3 (4)	O2J—N2J—C2J—C3J	8.6 (5)
C6D—C1D—C2D—C3D	−0.4 (6)	O1J—N2J—C2J—C3J	−172.2 (3)
N1D—C1D—C2D—N2D	2.0 (7)	O2J—N2J—C2J—C1J	−169.0 (3)
C6D—C1D—C2D—N2D	−179.0 (4)	O1J—N2J—C2J—C1J	10.2 (5)
O2D—N2D—C2D—C3D	3.1 (5)	N1J—C1J—C2J—C3J	−176.2 (4)
O1D—N2D—C2D—C3D	−177.9 (4)	C6J—C1J—C2J—C3J	2.2 (5)
O2D—N2D—C2D—C1D	−178.2 (4)	N1J—C1J—C2J—N2J	1.3 (6)
O1D—N2D—C2D—C1D	0.9 (6)	C6J—C1J—C2J—N2J	179.6 (3)
C1D—C2D—C3D—C4D	−0.4 (6)	N2J—C2J—C3J—C4J	−178.9 (3)
N2D—C2D—C3D—C4D	178.3 (3)	C1J—C2J—C3J—C4J	−1.3 (6)
C2D—C3D—C4D—C5D	0.6 (6)	C2J—C3J—C4J—C5J	−0.4 (6)
C2D—C3D—C4D—C7D	−179.4 (4)	C2J—C3J—C4J—C7J	179.8 (4)
C3D—C4D—C5D—C6D	0.0 (6)	C3J—C4J—C5J—C6J	1.2 (6)
C7D—C4D—C5D—C6D	−180.0 (4)	C7J—C4J—C5J—C6J	−179.0 (4)
C4D—C5D—C6D—C1D	−0.9 (6)	C4J—C5J—C6J—C1J	−0.3 (6)
N1D—C1D—C6D—C5D	−180.0 (4)	N1J—C1J—C6J—C5J	177.0 (4)
C2D—C1D—C6D—C5D	1.0 (6)	C2J—C1J—C6J—C5J	−1.4 (5)
C3D—C4D—C7D—O4D	−0.5 (6)	C3J—C4J—C7J—O4J	176.9 (4)
C5D—C4D—C7D—O4D	179.6 (4)	C5J—C4J—C7J—O4J	−2.9 (6)
C3D—C4D—C7D—O3D	−178.9 (4)	C3J—C4J—C7J—O3J	−2.5 (6)
C5D—C4D—C7D—O3D	1.1 (6)	C5J—C4J—C7J—O3J	177.7 (4)
C1D—N1D—C8D—C9D	−163.1 (5)	C1J—N1J—C8J—C9J	−161.9 (3)
N1D—C8D—C9D—C10D	−66.4 (6)	N1J—C8J—C9J—C10J	−64.6 (5)
C8D—C9D—C10D—C11D	126.3 (7)	C8J—C9J—C10J—C11J	111.6 (5)
C8E—N1E—C1E—C6E	−1.7 (6)	C8K—N1K—C1K—C6K	2.3 (6)
C8E—N1E—C1E—C2E	177.2 (4)	C8K—N1K—C1K—C2K	−177.6 (4)
N1E—C1E—C2E—C3E	−178.0 (4)	N1K—C1K—C2K—C3K	176.9 (4)
C6E—C1E—C2E—C3E	1.0 (6)	C6K—C1K—C2K—C3K	−3.0 (5)
N1E—C1E—C2E—N2E	0.5 (7)	N1K—C1K—C2K—N2K	−0.8 (6)
C6E—C1E—C2E—N2E	179.5 (4)	C6K—C1K—C2K—N2K	179.3 (3)
O2E—N2E—C2E—C3E	1.5 (6)	O2K—N2K—C2K—C3K	−1.8 (5)
O1E—N2E—C2E—C3E	−178.5 (4)	O1K—N2K—C2K—C3K	178.8 (3)
O2E—N2E—C2E—C1E	−177.0 (4)	O2K—N2K—C2K—C1K	176.1 (3)
O1E—N2E—C2E—C1E	3.0 (6)	O1K—N2K—C2K—C1K	−3.3 (6)
C1E—C2E—C3E—C4E	−0.4 (7)	C1K—C2K—C3K—C4K	0.3 (6)
N2E—C2E—C3E—C4E	−179.0 (4)	N2K—C2K—C3K—C4K	178.1 (3)
C2E—C3E—C4E—C5E	−0.1 (7)	C2K—C3K—C4K—C5K	2.2 (6)
C2E—C3E—C4E—C7E	179.4 (4)	C2K—C3K—C4K—C7K	−176.4 (4)
C3E—C4E—C5E—C6E	0.1 (7)	C3K—C4K—C5K—C6K	−1.9 (6)
C7E—C4E—C5E—C6E	−179.4 (4)	C7K—C4K—C5K—C6K	176.7 (4)
C4E—C5E—C6E—C1E	0.5 (7)	C4K—C5K—C6K—C1K	−0.9 (6)
N1E—C1E—C6E—C5E	178.0 (4)	N1K—C1K—C6K—C5K	−176.6 (4)
C2E—C1E—C6E—C5E	−1.0 (6)	C2K—C1K—C6K—C5K	3.3 (6)
C3E—C4E—C7E—O4E	3.9 (7)	C3K—C4K—C7K—O4K	−179.4 (4)
C5E—C4E—C7E—O4E	−176.6 (4)	C5K—C4K—C7K—O4K	2.0 (6)
C3E—C4E—C7E—O3E	−176.6 (4)	C3K—C4K—C7K—O3K	2.3 (6)
C5E—C4E—C7E—O3E	2.9 (6)	C5K—C4K—C7K—O3K	−176.2 (4)
C1E—N1E—C8E—C9E	−173.1 (4)	C1K—N1K—C8K—C9K	161.5 (4)
N1E—C8E—C9E—C10E	−58.3 (5)	N1K—C8K—C9K—C10K	62.6 (4)
C8E—C9E—C10E—C11E	123.7 (5)	C8K—C9K—C10K—C11K	−104.8 (5)
C8F—N1F—C1F—C2F	175.4 (4)	C8L—N1L—C1L—C6L	−1.0 (6)
C8F—N1F—C1F—C6F	−4.5 (6)	C8L—N1L—C1L—C2L	177.6 (4)
N1F—C1F—C2F—C3F	−179.0 (4)	N1L—C1L—C2L—C3L	−178.2 (4)
C6F—C1F—C2F—C3F	0.9 (6)	C6L—C1L—C2L—C3L	0.5 (5)
N1F—C1F—C2F—N2F	1.6 (7)	N1L—C1L—C2L—N2L	0.9 (6)
C6F—C1F—C2F—N2F	−178.5 (4)	C6L—C1L—C2L—N2L	179.6 (3)
O2F—N2F—C2F—C3F	4.1 (6)	O1L—N2L—C2L—C3L	−177.4 (4)
O1F—N2F—C2F—C3F	−175.4 (4)	O2L—N2L—C2L—C3L	2.6 (5)
O2F—N2F—C2F—C1F	−176.5 (4)	O1L—N2L—C2L—C1L	3.5 (6)
O1F—N2F—C2F—C1F	4.0 (6)	O2L—N2L—C2L—C1L	−176.5 (4)
C1F—C2F—C3F—C4F	−0.4 (7)	C1L—C2L—C3L—C4L	0.3 (6)
N2F—C2F—C3F—C4F	179.0 (4)	N2L—C2L—C3L—C4L	−178.8 (3)
C2F—C3F—C4F—C5F	0.5 (7)	C2L—C3L—C4L—C5L	−1.2 (6)
C2F—C3F—C4F—C7F	−179.3 (4)	C2L—C3L—C4L—C7L	179.2 (3)
C3F—C4F—C5F—C6F	−1.1 (7)	C3L—C4L—C5L—C6L	1.2 (6)
C7F—C4F—C5F—C6F	178.7 (4)	C7L—C4L—C5L—C6L	−179.2 (4)
C4F—C5F—C6F—C1F	1.7 (7)	C4L—C5L—C6L—C1L	−0.4 (6)
N1F—C1F—C6F—C5F	178.4 (4)	N1L—C1L—C6L—C5L	178.3 (4)
C2F—C1F—C6F—C5F	−1.5 (6)	C2L—C1L—C6L—C5L	−0.5 (6)
C3F—C4F—C7F—O4F	2.3 (7)	C3L—C4L—C7L—O4L	2.6 (6)
C5F—C4F—C7F—O4F	−177.5 (4)	C5L—C4L—C7L—O4L	−177.0 (4)
C3F—C4F—C7F—O3F	−176.8 (4)	C3L—C4L—C7L—O3L	−178.0 (4)
C5F—C4F—C7F—O3F	3.4 (6)	C5L—C4L—C7L—O3L	2.4 (5)
C1F—N1F—C8F—C9F	−161.5 (4)	C1L—N1L—C8L—C9L	−175.9 (4)
N1F—C8F—C9F—C10F	−65.7 (5)	N1L—C8L—C9L—C10L	−58.6 (5)
C8F—C9F—C10F—C11F	123.0 (5)	C8L—C9L—C10L—C11L	121.8 (6)

### Hydrogen-bond geometry (Å, °)

**Table:**

*D*—H···*A*	*D*—H	H···*A*	*D*···*A*	*D*—H···*A*
N1A—H1AA···O1A	0.86	2.00	2.643 (4)	130
N1B—H1BA···O1B	0.86	1.99	2.628 (5)	130
N1C—H1CA···O1C	0.86	1.98	2.631 (4)	132
N1D—H1DA···O1D	0.86	1.99	2.634 (5)	130
N1E—H1EA···O1E	0.86	2.01	2.637 (5)	129
N1F—H1FA···O1F	0.86	2.00	2.641 (4)	130
N1G—H1GA···O1G	0.86	1.98	2.624 (5)	130
N1H—H1HA···O1H	0.86	1.97	2.621 (5)	131
N1I—H1IA···O1I	0.86	1.99	2.632 (4)	130
N1J—H1JA···O1J	0.86	1.99	2.627 (4)	131
N1K—H1KA···O1K	0.86	1.96	2.615 (4)	132
N1L—H1LA···O1L	0.86	2.01	2.645 (4)	130
O3A—H3AB···O4C	0.82	1.80	2.612 (5)	176
O3C—H3CB···O4A	0.82	1.82	2.630 (4)	172
O3B—H3BB···O4L	0.82	1.81	2.626 (5)	173
O3L—H3LB···O4B	0.82	1.80	2.613 (5)	169
O3D—H3DB···O4J	0.82	1.80	2.618 (5)	173
O3J—H3JB···O4D	0.82	1.81	2.626 (5)	175
O3E—H3EB···O4I	0.82	1.81	2.624 (5)	170
O3I—H3IB···O4E	0.82	1.82	2.635 (5)	174
O3G—H3GB···O4K	0.82	1.80	2.616 (5)	172
O3K—H3KB···O4G	0.82	1.83	2.633 (5)	163
O3F—H3FB···O4H^i^	0.82	1.79	2.605 (5)	172
O3H—H3HB···O4F^ii^	0.82	1.82	2.611 (5)	164
C6B—H6BA···O2H^i^	0.93	2.45	3.169 (5)	134
C6H—H6HA···O2B^iii^	0.93	2.55	3.243 (5)	131
C6I—H6IA···O2C^ii^	0.93	2.53	3.218 (5)	131
C6K—H6KA···O2K^iv^	0.93	2.40	3.149 (4)	138
C9C—H9CB···O1B	0.97	2.59	3.477 (6)	152

Symmetry codes: (i) *x*−1/2, −*y*+1/2, *z*+1/2; (ii) *x*+1/2, −*y*+1/2, *z*−1/2; (iii) *x*+1/2, −*y*+1/2, *z*+1/2; (iv) *x*, −*y*+1, *z*−1/2.
